# A Homolog Pentameric Complex Dictates Viral Epithelial Tropism, Pathogenicity and Congenital Infection Rate in Guinea Pig Cytomegalovirus

**DOI:** 10.1371/journal.ppat.1005755

**Published:** 2016-07-07

**Authors:** Stewart Coleman, K. Yeon Choi, Matthew Root, Alistair McGregor

**Affiliations:** Department of Microbial Pathogenesis & Immunology, Texas A&M University, Health Science Center, College of Medicine, College Station, Texas, United States of America; University of California, Davis, UNITED STATES

## Abstract

In human cytomegalovirus (HCMV), tropism to epithelial and endothelial cells is dependent upon a pentameric complex (PC). Given the structure of the placenta, the PC is potentially an important neutralizing antibody target antigen against congenital infection. The guinea pig is the only small animal model for congenital CMV. Guinea pig cytomegalovirus (GPCMV) potentially encodes a *UL128-131* HCMV PC homolog locus (*GP128-GP133*). In transient expression studies, GPCMV gH and gL glycoproteins interacted with UL128, UL130 and UL131 homolog proteins (designated GP129 and GP131 and GP133 respectively) to form PC or subcomplexes which were determined by immunoprecipitation reactions directed to gH or gL. A natural GP129 C-terminal deletion mutant (aa 107–179) and a chimeric HCMV UL128 C-terminal domain swap GP129 mutant failed to form PC with other components. GPCMV infection of a newly established guinea pig epithelial cell line required a complete PC and a GP129 mutant virus lacked epithelial tropism and was attenuated in the guinea pig for pathogenicity and had a low congenital transmission rate. Individual knockout of *GP131* or *133* genes resulted in loss of viral epithelial tropism. A *GP128* mutant virus retained epithelial tropism and GP128 was determined not to be a PC component. A series of GPCMV mutants demonstrated that gO was not strictly essential for epithelial infection whereas gB and the PC were essential. Ectopic expression of a *GP129* cDNA in a *GP129* mutant virus restored epithelial tropism, pathogenicity and congenital infection. Overall, GPCMV forms a PC similar to HCMV which enables evaluation of PC based vaccine strategies in the guinea pig model.

## Author Summary

Congenital CMV is a leading cause of mental retardation and deafness in newborns. An effective vaccine against congenital CMV remains an elusive goal. HCMV encodes a pentameric glycoprotein complex (PC) necessary for tropism to epithelial, endothelial and myeloid cells. Given the structure of the placenta, the viral PC is considered important for congenital infection and potentially an important neutralizing antibody vaccine target antigen. The guinea pig, with a placenta structure similar to humans, is the only small animal model for congenital CMV. In this paper, GPCMV is shown to encode a homolog PC which enables epithelial tropism on a newly established cell line. It is likely that the GPCMV PC improves virus tropism to various cell types as PC positive virus has improved virus pathogenicity and congenital infection in vivo. This study lays important foundations for development of a PC based intervention strategy against congenital CMV in this model.

## Introduction

Human cytomegalovirus (HCMV or Human herpesvirus 5) is a member of the *Betaherpesvirinae* genus and encodes over 165 genes [1]. Viral infection is largely asymptomatic in healthy individual but establishes a lifelong mainly latent state in the host. However, infection of an immune compromised host (AIDS and transplant patients) or virus reactivation because of an impaired immune system can have severe consequences of morbidity or mortality [2, 3]. An additional important aspect of HCMV is congenital infection, where the virus crosses the placenta and infects the fetus in utero. This occurs in approximately less than 1% of live births [4] in the US and causes serious symptomatic disease including mental retardation and sensorineural hearing loss (SNHL) in newborns [4–8]. The greatest risk of congenital infection is to mothers who acquire a primary infection during pregnancy and prior immunity can reduce the risk by up to 69% [9]. Hence, the impact of a vaccine is potentially substantial, especially in countries where there is a greater risk of primary infection during pregnancy. These regions include the US, EU and Japan, where up to 50% of women of child bearing age are negative for HCMV [8, 10]. Licensed HCMV antivirals are available for transplant and AIDS patients but not congenital CMV [11]. Consequently, development of a vaccine against congenital CMV is a high priority.

Any proposed intervention for the prevention or treatment of HCMV infection should ideally be evaluated in a pre-clinical model. Unfortunately, HCMV is extremely species-specific. Consequently, animal model pathogenicity, vaccine and antiviral studies are carried out using animal-specific CMVs, including mouse, rat, guinea pig and rhesus macaques [12–16]. The guinea pig is unique insofar as it is the only small animal model to allow the study of congenital CMV infection, where the virus crosses the placenta and infects the fetus in utero unlike the mouse model [17]. Both human and guinea pig placentas are hemomonochorial containing a homogenous layer of trophoblast cells separating maternal and fetal circulation [18–20]. Additionally, as with human pregnancy, the gestation period (approximately 65 days) can be divided into trimesters. Importantly, GPCMV congenital infection causes disease in the fetus and in newborn pups similar to those found in humans including SNHL [21–23]. Consequently, the guinea pig model is best suited for testing of intervention strategies aimed at preventing congenital CMV infection [11, 24, 25].

A major drawback in GPCMV research has largely been overcome by the recent sequencing of the viral genome and the development of infectious BAC clones of GPCMV [15, 26–29]. Indeed, manipulation of GPCMV BACs has allowed the preliminary study of some viral genes [11, 30–36]. Additionally, the guinea pig animal genome has been sequenced (http://www.ensembl.org/Cavia_porcellus/Info/Index) which enables the development of new reagents for this model. Analysis of the GPCMV genome [15, 29] indicated that the virus encoded homologs to the HCMV glycoproteins (gB, gH, gL, gM, gN, gO) in genes co-linear with the HCMV genome (designated GP55, GP75, GP115, GP100, GP73 and GP74 respectively). In HCMV, these six glycoproteins (gB, gH, gL, gM, gN, gO) are required for fibroblast cell entry and they form the glycoprotein complexes, gCI (gB), gCII (gM/gN), gcIII (gH/gL/gO) on the viral membrane [37–39]. Additionally, in HCMV these complexes are important neutralizing antibody targets and as such potential vaccine candidates [40–44]. We recently demonstrated that GPCMV forms functionally similar glycoprotein complexes and these complexes are essential for infection of fibroblast cells as well as important target antigens [36]. In both HCMV and GPCMV, the viral glycoprotein gB is the immunodominant neutralizing viral antigen [45–49]. A recombinant HCMV gB has been investigated as a candidate subunit vaccine in phase II clinical trials but this provides at best approximately 50% efficacy despite high antibody titers [41].

The process of HCMV entry into the cell was assumed to occur via the mechanism of cell fusion mediated by gB but also requiring other glycoproteins [50–52]. Current studies indicate that it is the triplex (gH/gL/gO) and specifically gH/gL that promotes gB cell fusion in fibroblasts. This is in keeping with the general model for herpesviruses with core fusion being related to gB and gH/gL [53–58]. However, gM/gN is considered essential for virus infection of all cell types and is the most abundant complex in the virion [59]. Most of the earlier HCMV cell entry studies were performed on fibroblast cells with lab adapted strains of HCMV (eg. AD169). These viral strains lack the capacity to efficiently infect other cell types such as endothelial, epithelial cells. Clinical strains of HCMV also encode a pentameric glycoprotein complex (gH/gL/UL128/130/131) that enables viral entry into epithelial, endothelial and myeloid cells via an alternative pathway of cell entry that requires the pentameric complex (PC) in association with gB [55, 57, 60–66]. Alphaherpesviruses, unlike CMV, only encode one type of gH/gL complex. However, some gammaherpesviruses (eg. Epstein-Barr virus) encode two different gH/gL complexes to enable gB fusion into different cell types entry which potentially provides a model for CMV [54]. It is likely that gH/gL based complexes work upstream of gB for cell entry but this is poorly defined [58]. The alternative route of CMV cell entry will be referred to as the PC dependent pathway in this report. However, studies suggest that gO, which is unique to all CMV, also enhances infection of epi/endothelial cells by an undefined mechanism [67]. PC dependent virus infection of epithelial and endothelial occurs via a clathrin-independent endocytosis pathway with the endosomes undergoing an acid flux [61, 68]. PC dependent virus infection of dendritic cells is independent of pH but dependent upon cholesterol via macropinocytosis pathway [69]. Interestingly, a recent study demonstrated that virus entry into fibroblast cells can also occur via a pH/clathrin independent macropinocytosis pathway in virus devoid of the PC [70]. Undoubtedly, the PC is necessary for efficient entry into epithelial and endothelial cells. The viral locus encoding the PC unique genes (*UL128-131*) is unstable upon passage of clinical HCMV strains on fibroblast cells and encoded genes rapidly acquire point mutations or deletions with the subsequent loss of epi/endothelial viral tropism associated with the inability to form a functional PC [71]. Lab adapted strains of HCMV (eg. AD169) can infect epi/endothelial cells when the mutated locus is repaired or functional genes are expressed in an ectopic location which enable PC formation [64, 72, 73]. The basis for HCMV forming gH/gL/gO triplex or gH/gL PC is poorly defined but both complexes are present on the virion of clinical strains but ratios differ between strains [74]. Potentially, competitive binding of gO or UL128 with gH/gL might be a key stage but UL148 protein has also been suggested to play a role in the balance between these complexes [55, 75].

The PC is considered an important neutralizing target for HCMV on epithelial/endothelial cells and presumably for congenital infection, given the epi/endothelial structure of the placenta [76–78]. The importance of the PC as a target antigen was confirmed by the isolation of neutralizing human monoclonal antibodies to the PC which had higher potency than antibodies to other target antigens [76, 77]. In the context of congenital infection, high titer neutralizing antibodies are thought to be effective against transplacental viral transmission [76, 79, 80]. Potentially, a delay in the immune response to the PC results in fetal infection [81]. The importance of the PC in virus infection of cells is underscored by a recent finding for the gB subunit HCMV vaccine. In clinical trials, the gB vaccine induces a high titer neutralizing immune response which is effective in neutralizing virus on fibroblasts [41, 82]. However, in separate studies sera from gB vaccinated individuals is less effective at neutralizing virus infection on endothelial and epithelial cells in comparison to convalescent sera from HCMV infected individuals [78, 83, 84]. This demonstrated the importance of other viral neutralizing target antigens for infection on these cell types. Consequently, other target antigens should be considered important in the development of a vaccine against congenital CMV. Importantly, a gB vaccine fails to fully protect against congenital CMV in the guinea pig model [49, 85, 86].

Potentially, GPCMV encodes a homolog PC as a *UL128-131* homolog locus (*GP128-133*) was identified in low pass ATCC viral stock of GPCMV, strain 22122 (ATCC VR682) [28, 87]. The sequence of this virus also matched that of salivary gland (SG) GPCMV (extensively serially passaged in vivo in guinea pigs) [25]. However, a plaque purified isolate (designated PP ATCC), used to establish the first GPCMV sequence, carried a deletion in this locus [15]. PP ATCC had normal growth kinetics in tissue culture but was attenuated in vivo compared to SG GPCMV [25, 26]. We hypothesized that the viral attenuation was linked to the inability of the virus to form a homolog pentameric complex which impacted on virus cell tropism to specific cell types (eg. epithelial cells) and consequently pathogenicity in the animal. Virus cell tropism was evaluated with a newly established guinea pig renal epithelial cell line. The potential interactions of GPCMV homolog glycoproteins gH and gL with proteins encoded in the *GP128-133* locus were studied to provide evidence of a GPCMV PC. [63]. Additionally, a GPCMV mutant virus which encoded an intact locus but a mutated *GP129* gene (*UL128* homolog) was restored for epithelial tropism by expression of a full length *GP129* cDNA in an ectopic location. This recombinant virus (GP129FRT) incorporated a myc tagged GP129 into the viral particle as part of the PC. Knockout of *GP129*, *GP131* or *GP133* (*UL128*, *UL130* and *UL131* homologs respectively) in recombinant GPCMV impaired virus replication on epithelial cells but not fibroblasts. Importantly, virus restored for PC had improved pathogenicity and congenital transmission rates compared to mutant virus lacking the complex. Overall, the similarity of function between HCMV and GPCMV pentameric complexes strengthens the guinea pig model in the development of an effective preclinical vaccine strategy against epithelial and congenital infection based on the CMV PC.

## Results

### GPCMV epithelial tropism requires a full length *UL128-131* homolog locus

**Fig 1:**
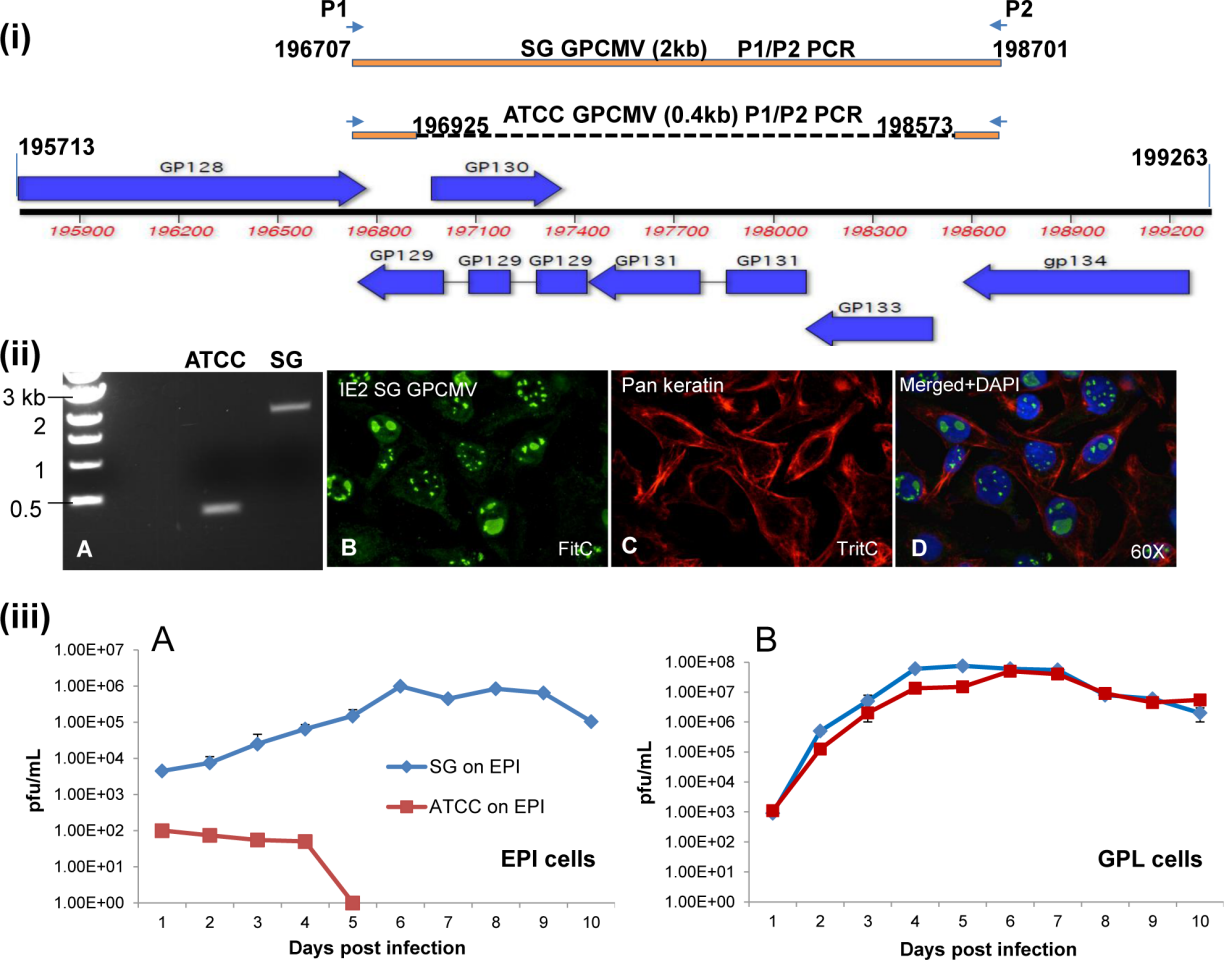
Structure of GPCMV *UL128-131* (*GP128-GP133*) homolog locus and wt or mutant virus growth on epithelial or fibroblast cells. **(i) Layout of the GPCMV *GP128-133* locus of genes.** Annotated GPCMV genome (co-ordinates 195,713–199,263 nucleotides) which encodes *GP128*-*gp134*. Individual genes represented as blue arrows and direction indicates either sense (above line) or complementary strand (below line) coding. Direct homologs to HCMV pentamer complex genes are *UL128 (GP129)*, *UL130 (GP131)*, *UL131 (GP133)*. *GP129* (3 exons) and *GP131* (two exons) are spliced genes. P1 (196,707) and P2 (198,701) show the location of the PCR primer pair (P1/P2) used to amplify the *GP128-GP133* locus genes. The top line shows amplification of the full length locus (2 kb) from SG GPCMV. The bottom line shows amplification of the deleted *GP128-GP133* locus in PP ATCC lab adapted virus (0.4 kb). Solid line indicates conserved sequence between full length locus and dotted line indicates deleted sequence. Co-ordinates 196,925–198,573 nucleotides represent the specific deletion within the locus for PP ATCC virus. Specific annotated nucleotide sequence of the *GP128-GP133* locus is shown in S1 Fig. **(ii) A.** Analysis of full length and truncated viruses and epithelial cell infection. Agarose gel electrophoresis of the P1/P2 PCR for the *GP128-133* locus from GPCMV. Left, kb ladder (Invitrogen). Middle, (ATTC) PP ATCC GPCMV (0.4 kb). Right, (SG) SG GPCMV (2 kb). **B, C and D.** Immunofluorescence assay of SG GPCMV infected epithelial cells. **B.** GPCMV IE2 detected with primary rabbit anti-IE2/ secondary anti rabbit IgG-FITC. **C.** GPCMV infected guinea pig epithelial cell monolayer verified by cytokeratin marker staining with primary mouse anti-pancytokeratin/ secondary anti-mouse IgG-TRITC. **D.** Overlay of B and C with cell nuclei stained with DAPI. **(iii)** Comparative growth curve of SG GPCMV and PP ATCC GPCMV on epithelial cells and fibroblast cells. Cells were infected with either at a moi of 1 pfu/cell. Sample were taken at different days post infection and titrated in duplicate on GPL cells as previously described [33]. Results plotted as virus titer against days post infection: A, growth on epithelial cells; B, growth on GPL cells. Diamond (blue), SG GPCMV. Square (red), PP ATCC.

In earlier reported studies of GPCMV, strain 22122 (ATCC VR682), the pathogenic salivary gland (SG) virus was maintained by serial passage in animals but could be attenuated by extensive serial passage of the virus on fibroblast cells, >11–25 passes [21, 88, 89]. However, the molecular basis for this attenuation was undefined and the viral stocks of the SG and fibroblast passaged virus are no longer available (Griffith (Yale University, CT) personal communication to AM). A potential basis for the viral attenuation in GPCMV might be by modification of a homolog *UL128-133* locus [28, 87, 90]. In clinical HCMV strains, adaptation of the virus to growth on fibroblast cells rapidly resulted in mutations in this locus and impaired virus tropism to various cell types [60, 71, 91]. Inoue and colleagues [87] identified two variants of GPCMV in low pass ATCC stock of GPCMV (strain 22122). One GPCMV variant was intact for the homolog *UL128-131* locus (*GP128-GP133*), whereas the other carried a deletion in the locus and was similar in sequence to the tissue culture adapted GPCMV isolate [15, 29]. GPCMV, strain 22122 (ATCC), was serially propagated in guinea pigs at Children’s Hospital Research Foundation, Cincinnati (Ohio, USA) from the late 1980s-2005. Salivary gland (SG) viral stocks were extensively used in congenital GPCMV challenge studies by a number of investigators. This SG virus stock was recently sequenced and shown to encode a full length *GP128-133* locus [92], whereas the tissue culture adapted virus derived from the SG GPCMV stock used to establish the first viral genome sequence carried a deletion in this locus [15]. In this present study, GPCMV salivary gland stock SG11 (11 direct serial passages in guinea pigs) was used to evaluate the sequence of the *GP128-133* locus in the virulent virus. Additionally, plaque purified (PP ATCC) virus stock extensively passaged on fibroblast cells was used to evaluate the sequence of lab adapted virus. PCR primers ([87], S1 Table) were used to amplify the *GP128-GP133* locus and the PCR product cloned prior to sequencing. Fig 1(i) shows the structure of the *GP128-133* locus and the analysis of the cloned PCR products for the *GP128-133* locus from respective viruses. Sequence analysis of the cloned PCR products (see Fig 1(ii)A and S1 Fig) confirmed that the two virus stocks (SG GPCMV and PP ATCC plaque isolate) differed in an identical fashion to the two isolates reported in low pass ATCC stock [28, 87]. The SG GPCMV had a complete *GP128-133* locus (2 kb PCR product), whereas the PP ATCC virus [26] carried a 1.6 kb deletion (0.4 kb PCR product) which removed the majority of the *GP129-133* coding sequence (196,925–198,573) as shown by PCR analysis (see Fig 1(ii)A). This potentially supports the hypothesis that a full length *GP128-133* locus is necessary for full tropism/pathogenicity in vivo since GPCMV mutated in the *GP128-133* locus is attenuated in the animal model [29]. RT-PCR at late stage infection confirmed transcription of genes encoded in the *GP128-133* locus in SG GPCMV infected cells (S2 Fig).

In HCMV, a consequence of adaptation of clinical strain virus to fibroblast cells is an inability for the virus to form a functional PC (gH/gL/UL128-131) necessary for infection of epithelial or endothelial cells and other cell types [60, 62–64, 71]. Potentially, GPCMV encodes a homolog PC but cellular tropism associated with this locus has not been successfully demonstrated [90] except for infection of macrophage [93]. A major limitation in GPCMV studies is the availability of different types of tissue culture cell lines to evaluate virus tropism. Consequently, we generated a novel guinea pig renal epithelial cell line from the kidney. Colonies were clonally isolated, characterized by cytokeratin marker and cell lines immortalized as described in materials and methods. Epithelial cells were characterized by assay for cytokeratin, either by western blot or immunofluorescence assay (see S3 Fig). The guinea pig epithelial cells were positive for cytokeratin unlike GPL cells (S3 Fig). Importantly, SG GPCMV was capable of infecting and replicating on epithelial cells. Fig 1(ii) shows virus infected epithelial cells co-stained for cytokeratin (cytoplasm) and GPCMV IE2 protein (nucleus), see panels C and B respectively. In contrast, viral antigens failed to be detected in PP ATCC GPCMV infected epithelial cells (S4 Fig) but control studies demonstrated virus infection of fibroblast cells (S4 Fig). Additionally, a growth curve of SG GPCMV vs PP ATCC demonstrated that virus with an intact *GP128-133* locus can infect and replicate on epithelial cells, whereas the PP ATCC mutant virus was highly impaired for growth on epithelial cells, see Fig 1(iii)A. In contrast, both viruses were capable of normal growth on fibroblast cells (Fig 1(iii)B).

It was concluded that the SG GPCMV has the ability to replicate on epithelial cells, unlike lab adapted GPCMV. Additionally, tropism was a result of a SG GPCMV virus encoding a complete *GP128-133* locus unlike lab adapted virus. This could be verified by comparative sequence analysis between isolates which indicated that the only difference between viruses related to the *GP128-133* locus [29, 87, 94].

### Pentameric glycoprotein complex formation and impact of GP129 mutants

In order to study GPCMV pentameric glycoprotein complex formation, full length cDNA clones of *GP128*, *GP129*, *GP131* and *GP133* were cloned into mammalian expression vectors or recombinant adenovirus vectors under HCMV MIE promoter control as described in materials and methods [36]. Additionally, ORFs were C-terminal epitope tagged to enable detection: GP129 (myc); GP131 (HA); GP128 (FLAG); and GP133 (FLAG). Also, previously described GP75 (gH ORF) and GP115 (gL ORF), C-terminal tagged with GFP or mCherry respectively, in mammalian expression plasmids or recombinant defective adenovirus [36] were utilized in the study. All constructs were verified by sequencing and are summarized in S5 Fig.

**Fig 2:**
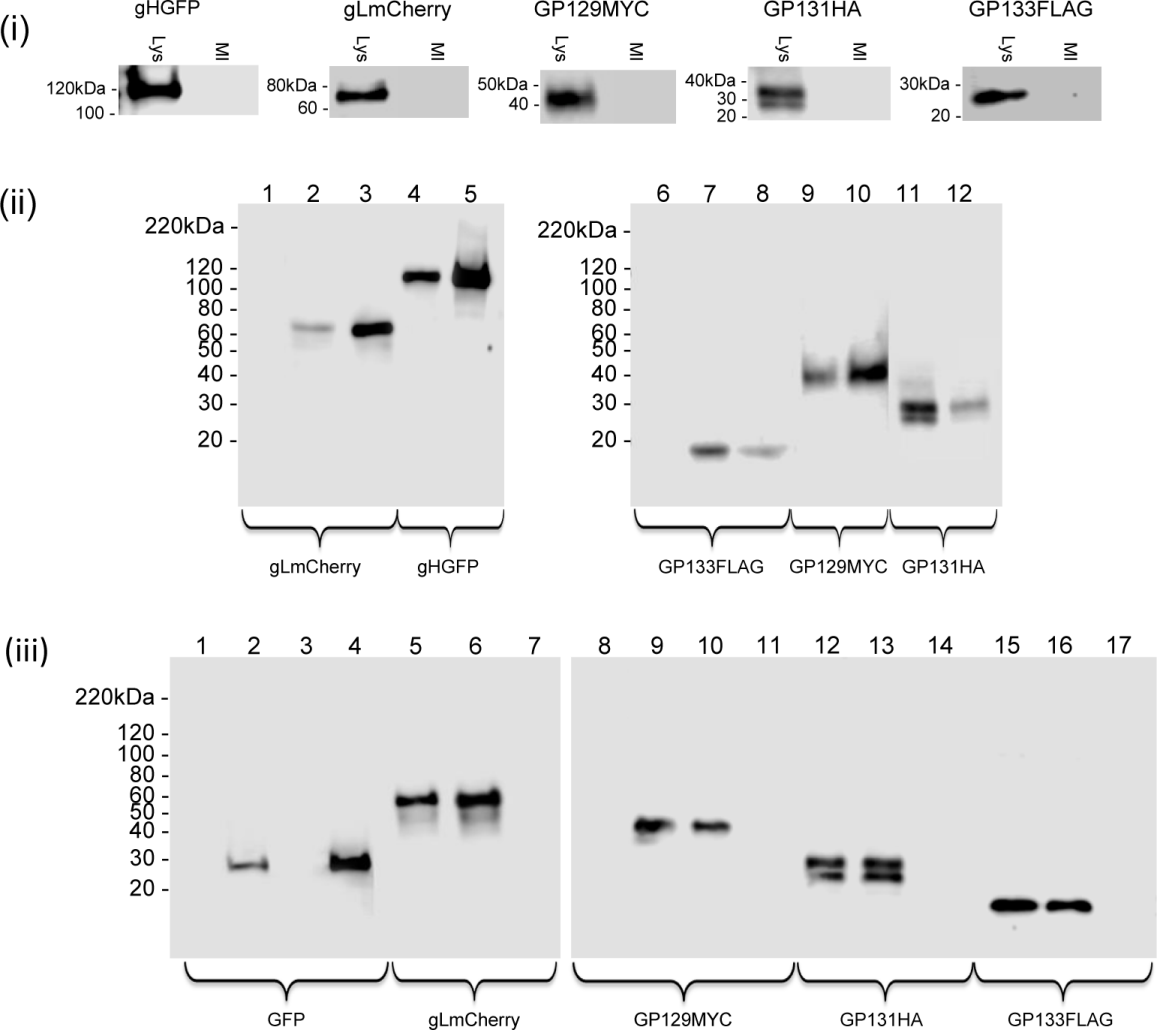
Immunoprecipitation of GPCMV pentameric complex. **(i)** Individual expression of pentameric complex components in epithelial cells transduced with recombinant adenovirus encoding either gHGFP, gLmCherry, GP129myc, GP131HA or GP133FLAG analyzed by western blot using respective primary antibodies: gH (anti-GFP); gL (anti-mCherry); GP129 (anti-myc); GP131 (anti-HA); GP133 (anti FLAG). Bands visualized with appropriate secondary antibody-HRP conjugate as described in materials and methods [36]. Lys indicates defective adenovirus transduced lane and MI indicates mock cell lysate. **(ii)** Immunoprecipitation of the pentameric complex from epithelial cells transduced with all five recombinant defective adenoviruses encoding individual components (10 TDU/virus/cell). Samples were analyzed as total cell lysate as above or processed for immunoprecipitation assay. Immunoprecipitation was carried out with GFP trap as previously described [36]. Individual proteins were detected as described for (i). Western blot lanes: 1–3 (anti-mCherry); 4–5 (anti-GFP); 6–8 (antiFLAG); 9–10 (anti-myc); 11–12 (anti-HA). Lanes 1 and 6 mock infected cell lysate. Lanes 2, 4, 7, 9 and 11 (total cell lysate). Lanes 3, 5, 8, 10 and 12 (immunoprecipitation). **(iii)** Control PC immunoprecipitation assay with GFP substituting for gHGFP. Epithelial cells transduced with defective recombinant adenoviruses encoding GFP, gLmCherry GP129myc, GP131HA and GP133FLAG followed by GFP immunoprecipitation and western blot for proteins as previously described [36]. Western blot lanes: 1–4 (anti-GFP); 5–7 (anti-mCherry); 8–11 (anti-myc); 12–14 (anti-HA); 15–17 (anti-FLAG). Lanes 1 and 8 mock infected cell lysate. Lanes 2, 5, 9, 12 and 15 (total cell lysate). Lanes 3, 6, 10, 13 and 16 (flow through). Lanes 4, 7, 11, 14 and 17 (immunoprecipitation).

An initial series of experiments examined the cellular localization of the various GPCMV proteins. Cells transduced with defective adenoviruses expressing gH, gL, GP129, GP131 and GP133 resulted in a cytoplasmic co-localization of gH and gL with GP129, GP131 and GP133 (S5 Fig). Next, an immunoprecipitation assay was performed on cells that expressed all the components of the potential pentameric complex to demonstrate protein:protein interactions. Fig 2(i) demonstrated that transduction of epithelial cells with recombinant adenovirus encoding a single glycoprotein enabled their successful detection by western blot analysis using their respective epitope tag marker. Transient expression of gHGFP and gLmCherry have previously been described [36]. Western blot analysis of transiently expressed GP129myc, GP131HA and GP133FLAG produced proteins with larger than expected size: GP129myc (expected 24 compared to 40kDa); GP131HA (25 compared to 31 kDa); GP133FLAG (16.7 compared to 19 kDa) (S2 Table). It was presumed that this was a result of post-translation modification such as glycosylation as previously demonstrated for gH and gL [36]. Potentially, both glycosylated and non-glycosylated versions of GP131HA are seen in Fig 2(i) as proteins at two different molecular weights (approximately 31 and 25 kDa) are detected. Treatment of cells with the glycosylation inhibitor tunicamycin (S6 Fig) demonstrated that GP129 and GP131 were subject to glycosylation as predicted in S2 Table. In the presence of tunicamycin, only lower molecular weight proteins were detected. Tunicamycin treatment also resulted in proteins appearing more aggregated in cellular immunofluorescence studies (S6 Fig).

Next, GFP-trap (Chromotek) immunoprecipitation (IP) assays were used to detect interaction with gH-GFP/ PC formation in cells transduced by all five recombinant adenoviruses (AdgHGFP, AdgLmCherry, AdGP129myc, AdGP131HA and AdGP133FLAG), Fig 2(ii). The GFP trap approach had previously been used to successfully study GPCMV glycoprotein complexes gM/gN (via gMGFP IP) and gH/gL/gO (via gHGFP IP) [36]. The GFP-trap IP of lysed cells transduced for all potential PC protein components resulted in the successful IP of gLmCherry, GP129myc, GP131HA and GP133FLAG by gHGFP (Fig 2(ii)). Interestingly, both species of GP131HA were immunoprecipitated. In contrast, a control GFP trap IP of cells transduced with AdGFP, AdgLmCherry, AdGP129myc, AdGP130HA and AdGP133FLAG but not AdgHGFP resulted in successful IP of GFP but none of the components of the PC (Fig 2(iii)), which demonstrated the specificity of the IP assay as well as the importance of gH in the interactions with other PC proteins. A similar series of PC immunoprecipitation reactions were also performed using RFP-trap (Chromotek) with gLmCherry and control mCherry. All PC components were able to be precipitated by gLmCherry IP which demonstrated the importance of gL for PC formation (S7 Fig). A control mCherry in place of gLmCherry failed to immunoprecipitate components of the PC (S7 Fig)

**Fig 3:**
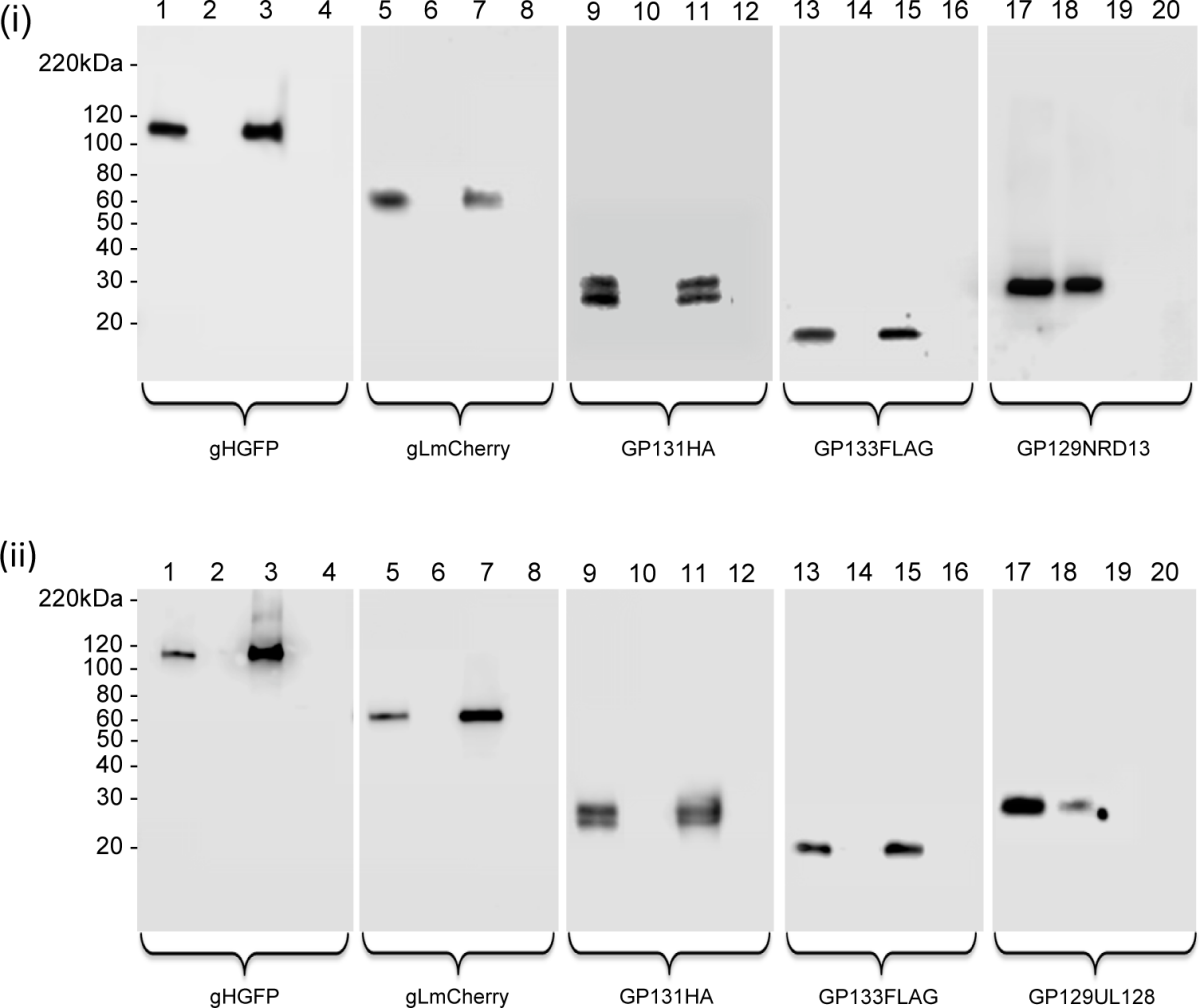
PC formation and GP129 mutants. C-terminal GP129 mutants were evaluated for an ability to form pentamer complexes with gHGFP, gLmCherry, GP131HA and GP133FLAG. GP129 mutants were expressed on transient expression plasmids transfected onto epithelial cells. Immunoprecipitation was carried out using a GFP trap as described in materials and methods. GP129 mutants (NRD13 and GP129UL128) were detected by a myc epitope tag. **(i)** PC formation assay using gHGFP and other components of the PC and GP129 mutant (NRD13) and GFP trap IP. Lanes 1, 5, 9, 13 and 17 are total cell lysates analyzed by respective antibodies. Lanes 3, 7, 11, 15 and 19 are immunoprecipitation tracks using GFP trap [36]. Lanes 2, 6, 10, 14, 18 are flow through wash tracks prior to immunoprecipitation. Lanes 4, 8, 12, 16 and 20 are mock cell lysate. Samples were assayed for: gH (anti-GFP), lanes 1–4; gL (anti-mCherry), lanes 5–8; GP131 (anti-HA), lanes 9–12; GP133 (anti-FLAG), lanes 13–16; and GP129 mutant NRD13 (anti-myc), lanes 17–20. Secondary antibody was anti-mouse IgG-HRP. **(ii)** PC formation assay using gHGFP and other components of the PC and GP129 mutant (GP129UL128) and GFP trap IP. Lanes as described for (i) except lanes 17–20 GP129UL128 mutant blot.

**Fig 4:**
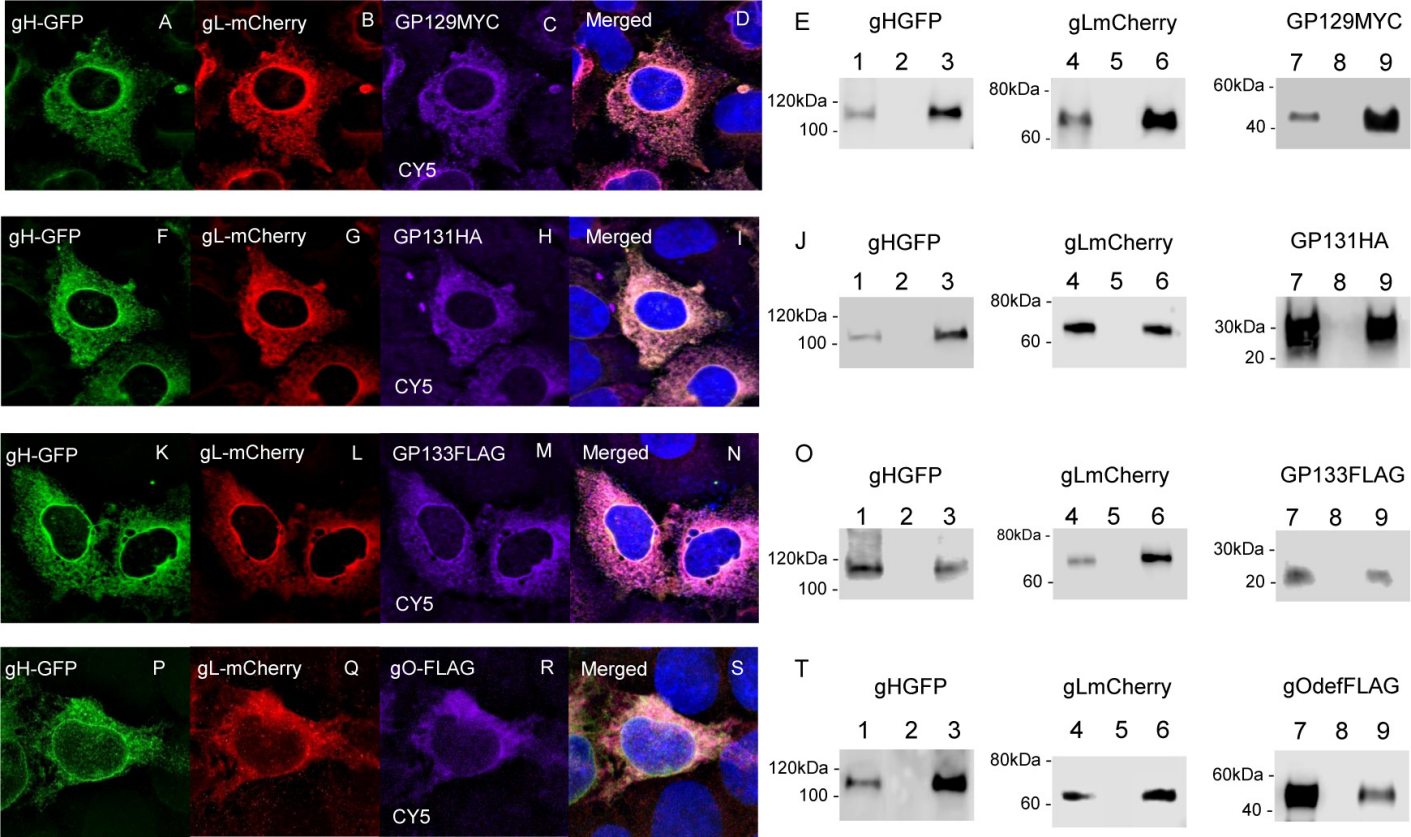
gH/gL triplex formation with GP129, GP131, GP133 or gO. GP129, GP131, GP133 and gO were evaluated for an ability to form triplex complexes with gH and gL. Transient expression of epithelial cells with gHGFP, gLmCherry, GP129myc, GP131HA and GP133FLAG was as described in materials and methods. Evaluation of triplex formation was by cellular colocalization or by GFP trap immunoprecipitation assay. **A-E.** gHGFP/gLmCherry/GP129myc triplex formation. Panels A-D, transient expression of individual proteins in epithelial cells and co-localization shown in merged image (D). Western blot of triplex immunoprecipitation (E). Lanes 1, 4 and 7 (total cell lysate). Lanes 3, 6 and 9 (IP). Lanes 2, 5 and 8 (flow through wash). **F-J.** gHGFP/gLmCherry/GP131HA triplex formation. Panels F-H, transient expression of individual proteins in epithelial cells and co-localization shown in merged image (I). Western blot of triplex immunoprecipitation (J) as described for E except lanes 7–9 GP131HA western. **K-O.** gHGFP/gLmCherry/GP133FLAG triplex formation. Panels K-M, transient expression of individual proteins in epithelial cells and co-localization shown in merged image (N). Western blot of triplex immunoprecipitation (O) as described for E except lanes 7–9 GP133FLAG western. **P-T.** gHGFP/gLmCherry/gOFLAG triplex formation. Panels P-R, transient expression of individual proteins in epithelial cells and co-localization shown in merged image (S). Western blot of triplex immunoprecipitation (T) as described for E except lanes 7–9 gOdelFLAG. Cellular co-localization merged figures (D, K, N and S) include DAPI co-stained cells. GP129myc, GP131HA, GP133FLAG and gOdelFLAG detected by primary anti-epitope antibody and secondary anti-mouseIgG-Cy5 (immunofluorescence) and anti-mouseIgG-HRP (western blot). Both gHGFP and gLmCherry were detected by fluorescence (cell localization) and specific epitope antibody (western). Panels E, J, O and P western blots. Lanes: 1, 4 and 7 total cell lysate; 2, 5 and 8 wash flow through; 3, 6 and 9 immunoprecipitation.

Next, we investigated the impact of C-terminal GP129 (UL128 homolog) mutants on the ability to form a pentameric complex with other GPCMV proteins. An initial study was carried out with a natural GP129 mutant. The second generation GPCMV BAC derived virus encodes the full spectrum of viral genes [29, 95] but contains a 4 bp deletion in the *GP129* gene, which places the ORF out of frame and truncated the encoded protein at codon 102 (NRD13, see S8 Fig). Virus derived from this BAC has normal growth kinetics on fibroblast cells but lacked the ability to grow on epithelial cells (see section on restoration of epithelial tropism). A cDNA clone of the truncated GP129 mutant (designated GP129NRD13) was myc-epitope tagged and cloned into a transient expression vector and assayed for an ability to form a PC in a GFP trap immunoprecipitation assay. Despite detectable GP129NRD13 expression levels, the protein failed to be immunoprecipitated as part of a PC (Fig 3). This indicated the importance of the C-terminal domain of GP129 in complex formation. A chimeric GP129 C-terminal mutant was also generated synthetically that encoded the C-terminal domain of HCMV UL128 (Merlin strain) in place of GP129 after the NRD13 truncation site (designated GP129UL128, see S8 Fig). In transient expression studies, the GP129UL128 chimeric protein failed to form a PC with other components (Fig 3). This indicated that there was insufficient conservation between the C-terminal domains of GPCMV GP129 and HCMV UL128 to enable PC formation. Although the GP129 mutants could not be precipitated as part of a PC, the gHGFP immunoprecipitation reactions did pull down other components of the complex (gL, GP131 and GP133). Potentially, components of the PC were capable of forming subcomplexes with gH/gL. Consequently, the ability of GP129, GP131 and GP133 to independently form triplexes with gH/gL was investigated. In transient expression immunoprecipitation assays based on gHGFP, gH/gL formed triplex complexes with GP129, GP131 or GP133 (Fig 3). Protein cellular co-localization could also been demonstrated in the cytoplasm of epithelial cells (Fig 4 and S9 Fig). The relevance of the various subcomplex triplexes to the PC or to the gH/gL/gO triplex formation and viral assembly remains to be more fully investigated in future studies but would indicate that gH/gL interaction is not solely dependent upon GP129. Subcomplex formation was also investigated for the GP129 mutant. Triplex formation could not be demonstrated to occur with gH, gL and GP129 NRD13 with a similar outcome to that obtained for interaction with all PC components (Fig 3). This implied that the C-terminal portion of GP129 was also important for subcomplex formation. Overall, it was concluded that GPCMV forms a homolog pentameric complex and that the UL128 homolog (GP129), especially the C-terminal domain is important for complex formation.

### Restoration of epithelial tropism in lab adapted GPCMV and requirement for components of the *GP128-133* locus

**Fig 5:**
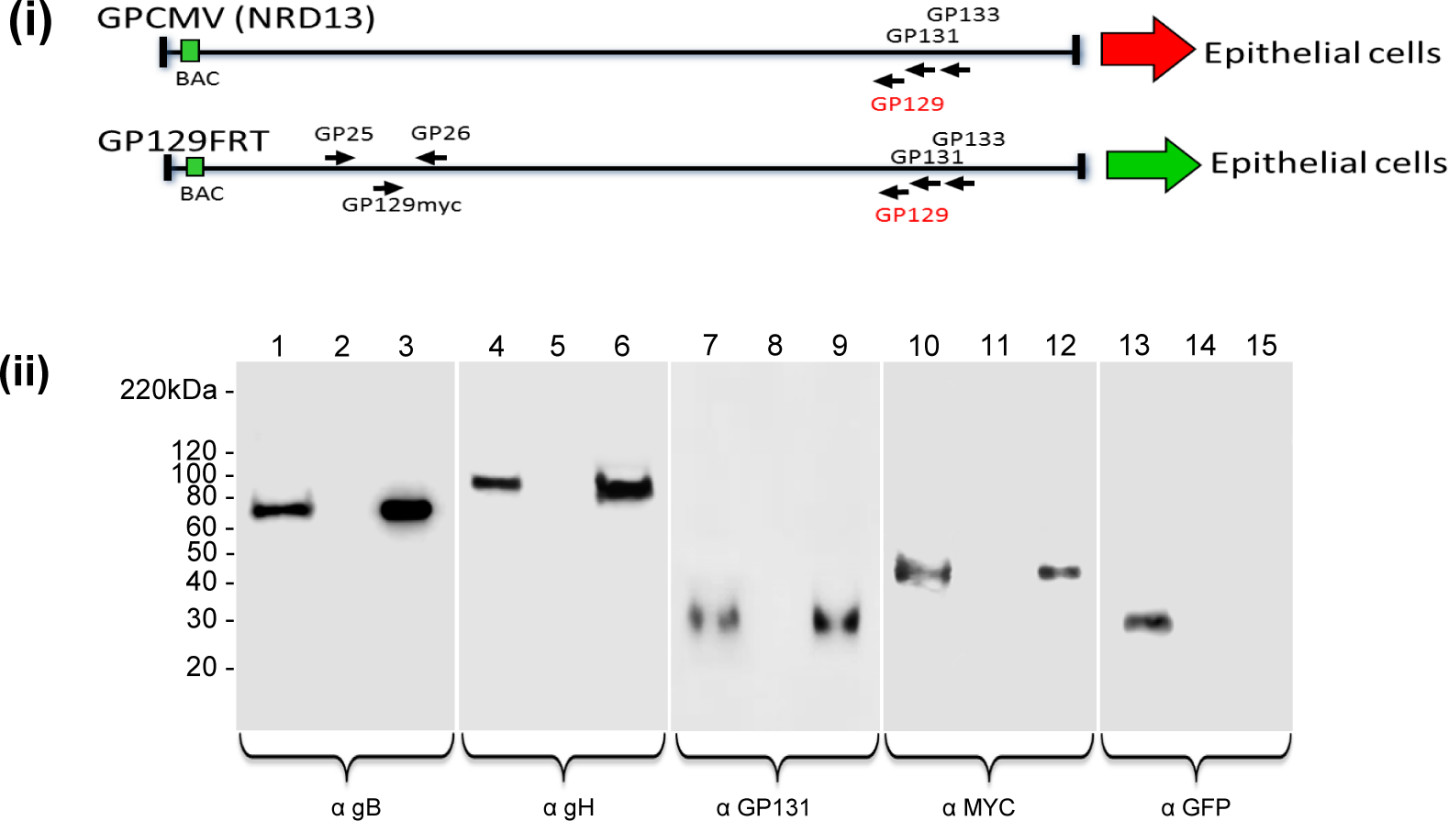
Ectopic expression of GP129 and incorporation into the virion. **(i)** Structure of parental BAC virus (NRD13) and modified mutant GP129FRT GPCMV. Modified mutant encodes an ectopic GP129 (myc tagged) cDNA under SV40 promoter control in the *GP25/GP26* intergenic locus as described in materials and methods (S14 Fig). Arrows (right) indicate virus tropism to epithelial cells: red, no tropism; green, tropism. **(ii)** Western blot analysis of sucrose gradient purified GP129FRT. Purified virus was evaluated for the presence of structural proteins present in the viral membrane by western blot analysis: gB (detected by mouse anti-gB), lanes 1–3; gH (rabbit anti-gH), lanes 4–6; GP131 (mouse anti-GP131), lanes 7–9; GP129 (mouse anti-myc), lanes 10–12. Additionally, a control GFP protein (mouse anti-GFP) expressed by the virus was also evaluated (lanes 13–15). Secondary antibodies were either anti-mouse IgG/HRP or anti-rabbit IgG/HRP. Lanes: 1, 4, 7, 10 and 13 total cell lysate (GP129FRT); 2, 5, 8, 11 and 14 total cell lysate (uninfected); 3, 6, 9, 12, and 15 (purified virus particle). Equivalent protein loading was determined by Bradford assay.

**Fig 6:**
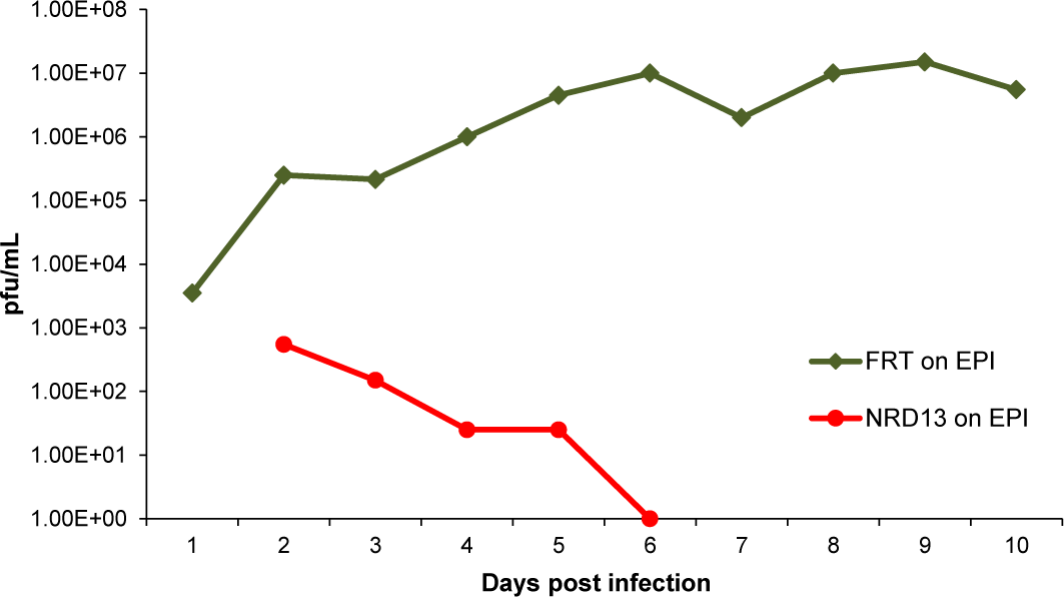
Comparative growth kinetics of GP129FRT and NRD13 GPCMV on epithelial cells. Guinea pig epithelial cells were infected with either GP129FRT (encoding an ectopic GP129) or NRD13 (encoding a mutant GP129) at a moi of 1 pfu/cell. Sample were taken at different days post infection and titrated in duplicate and titrated on GPL cells as previously described [33]. Results plotted as virus titer against days post infection.

GPCMV serially passaged in animals as salivary gland stock retained an ability to infect epithelial cells unlike lab adapted virus. The second generation GPCMV BAC encoded a full length *GP128*-*GP133* locus but carried a 4 bp deletion that truncated the GP129 ORF (NRD13 mutant, S8 Fig) compared to wild type virus [29, 95]. Transient expression of GP129NRD13 protein together with other components of the pentameric complex (gH, gL, GP131 and GP133) failed to generate a detectable PC (Fig 4). Additionally, BAC derived GPCMV lacked epithelial tropism but grew normally on fibroblast cells (see below) [96]. The capability of BAC derived GPCMV to form a functional pentameric complex and potentially restore epithelial tropism was evaluated by the introduction of a full length *GP129* cDNA expression cassette into the GPCMV genome in a non-essential intergenic locus. The intergenic site between *UL25* and *UL26* homologs *(GP25* and *GP26)* was selected on the basis of co-terminal transcripts ending in this locus with sufficient intergenic sequence to enable insertion of an ectopic cassette without interfering with GP25 or GP26 expression based on previous studies [25] (see S10 Fig). A cDNA myc tagged GP129 ORF used in transient expression studies was cloned initially into a shuttle vector (pGP2526GP129LinkKm) which placed the *GP129* cDNA under SV40 promoter and SV40 polyA control as described in materials and methods (S11 Fig). Mutant GPCMV BAC clones were selected by kanamycin (Km) marker. Full length GPCMV BAC mutants encoding GP129myc in the *GP25/GP26* locus were verified by restriction enzyme profile analysis (S12 Fig) and by PCR (S11 Fig) and sequencing. DNA from correctly identified mutant GPCMV BACs were transfected onto GPL cells to generate virus (GP129FRT). Virus expression of myc tagged GP129 and protein incorporation into the virus particle was demonstrated by western blot analysis of sucrose gradient purified viral particles. Fig 5 demonstrated that GP129myc was expressed in virus infected cells. Additionally, that GP129 was present in the purified viral particles as was GP131, another unique component of the PC. Glycoprotein gH could also be detected in virus particles, presumably as part of the triplex homolog (gH/gL/gO) and also the pentameric complex (gH/gL/GP129/GP131/GP133). Additionally, gB glycoprotein could be detected as a viral particle component but not GFP, which was expressed in infected cells but not incorporated into the viral particle. A growth curve confirmed that GP129FRT GPCMV was highly trophic for epithelial cells with efficient virus growth (Fig 6). In comparison, the parental derived GPCMV BAC virus NRD13 which encoded a truncated GP129 but viable GP131 and GP133 failed to efficiently replicate on epithelial cells (Fig 6).

**Fig 7:**
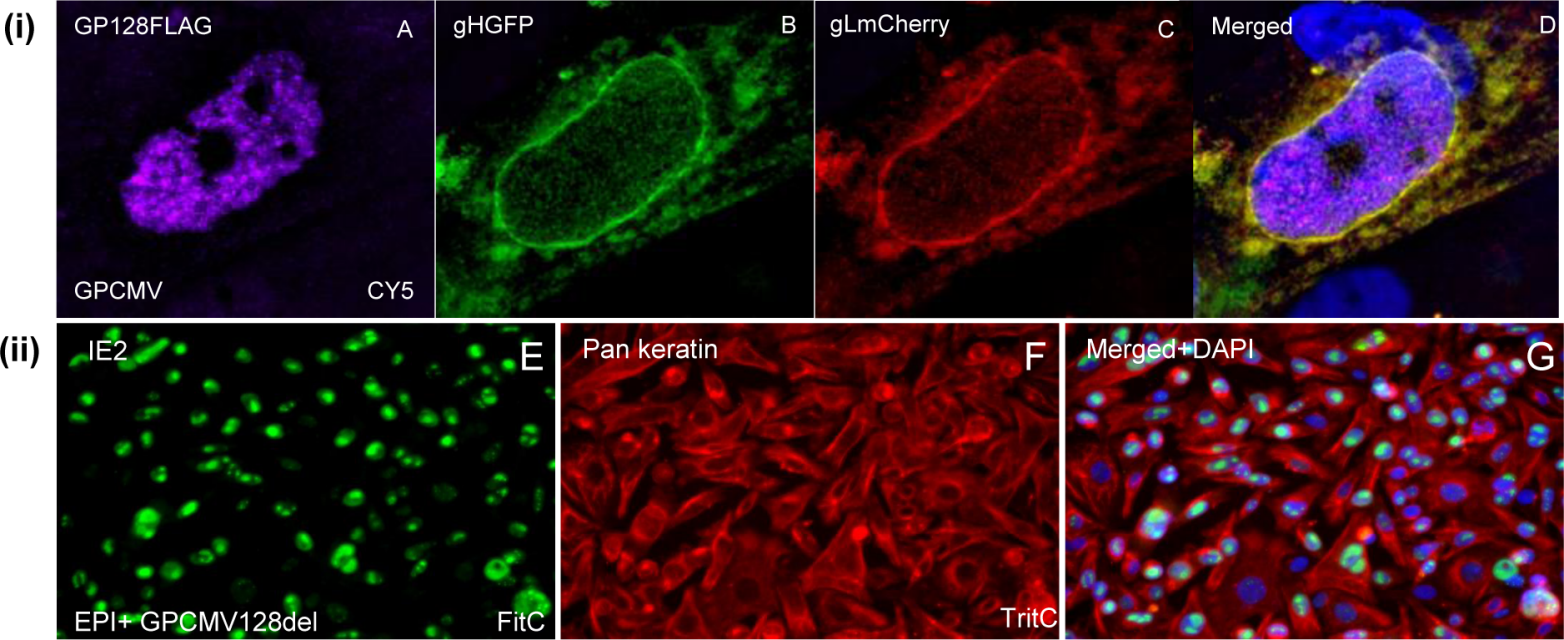
GP128 is not a component of the pentameric complex and a *GP128* mutant is not impaired for epithelial cell tropism. **(i)** GP128 is a nuclear protein. Transient plasmid co-expression of GP128 (FLAG tagged), gHGFP and gLmCherry at 24 hours post transfection. Panels A-D same cell. Panel A, immunofluorescence of GP128FLAG (mouse anti-FLAG/ anti-mouse IgG-Cy5); GFP fluorescence of gHGFP (B); mCherry fluorescence of gLmCherry (C); merged images A-C (D). **(ii)** GP128 knockout virus (GPCMVdel128) grows on epithelial cells. E. Immunofluorescence of IE2 (rabbit anti-IE2/ anti-rabbit IgG-FITC). F. Immunofluorescence of cytokeratin (mouse anti-pancytokeratin/anti-mouse IgG-TRITC). G. Merged image of panels A plus B and DAPI stained cell nuclei.

Next, the requirement for other components of the homolog PC for virus growth on epithelial cells was evaluated. Targeted mutagenesis was performed on the *GP128-GP133* locus to generate GPCMV BAC mutants with knockout *GP128*, *GP129*-*GP131* or *GP133* in separate BAC mutagenesis reactions. Final GPCMV BAC mutants encoded an ectopic *GP129* cDNA in the *GP25/GP26* intergenic locus in addition to specific mutations in the *GP128-GP133* locus as described in materials and methods. BAC mutants were characterized by restriction profile analysis, PCR and sequencing of the PCR product (S12 Fig and S11 Fig). Recombinant viruses were designated GP128FRT/GP129Link (*GP128* mutant); GP129-GP131FRT/GP129Link (*GP131* mutant); GP133FRT/GP129Link (*GP133* mutant). Although these viral mutants had similar growth kinetics on GPL cells, they lacked an ability to efficiently infect epithelial cells (S13 Fig). The exception was the *GP128* mutant which retained an ability to infect epithelial cells (Fig 7). In the case of *GP131* and *GP133* GFP tagged mutant viruses (non-cre BAC excised) a contrasting infection could be demonstrated between fibroblast and epithelial cells. In separate experiments, a moi of 1 pfu/cell resulted in a 100% infection of GPL cells but only approximately 1 in 10^6^ epithelial cells. Additionally, mutant virus infected epithelial cells failed to result in virus spread to surrounding cells, unlike GP129FRT or SG GPCMV. Examples of impaired *GP131* and *GP133* mutant virus growth on epithelial cells compared to GPL cells is shown in S13(ii) Fig.

Overall, it was concluded that epithelial tropism could be restored to lab adapted GPCMV by ectopic expression of a missing full length GP129 protein. This virus (GP129FRT) expressed GP129 and GP131 proteins as part of the viral particle. Knockout of individual genes in the *GP128-GP133* locus in the backdrop of virus expressing the ectopic full length GP129 also confirmed the essential role of GP131 and GP133 in PC formation and epithelial tropism. Knockout of the *GP128* gene did not prevent epithelial tropism and transient expression of GP128 demonstrated that it was a nuclear targeting protein (Fig 7). The functional significance of GP128 in the GPCMV life cycle remains unknown. BLAST analysis of the predicted GP128 protein sequence indicated that it was a potential homolog of MCMV IE2 [15] and therefore is unlikely to be relevant to PC formation.

### Alternative pathway of cell entry can be demonstrated in GPCMV by elimination of glycoprotein triplex formation (gH/gL/gO) and retention of glycoprotein PC formation

**Fig 8:**
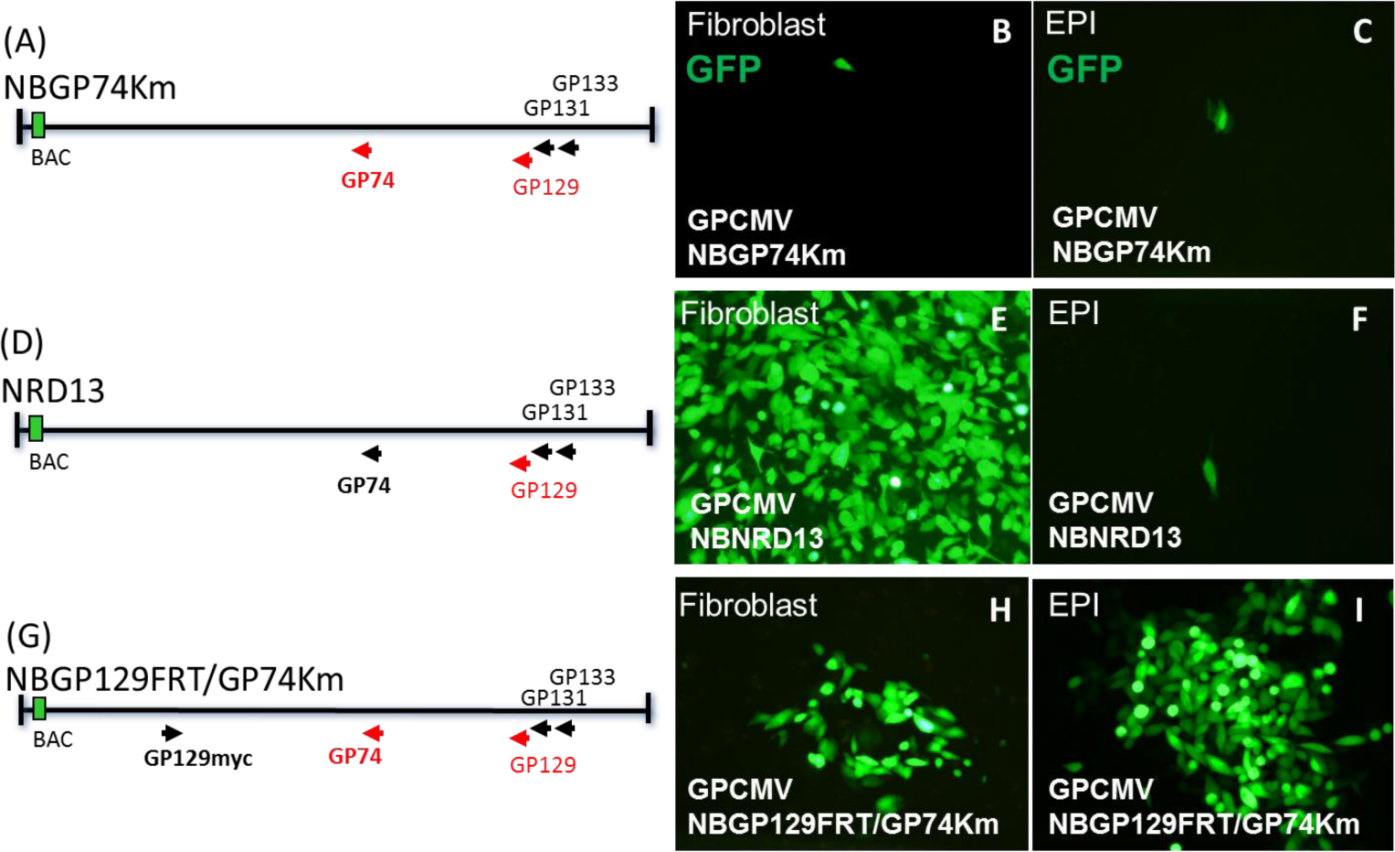
PC+/gO negative GPCMV demonstrates that infection of epithelial cells is independent of the gH/gL/gO triplex. GPCMV BAC mutants were transfected onto cells (GPL and EPI) to generate GFP positive virus and to demonstrate a requirement for the pentameric complex but not the triplex for epithelial cell tropism. GPCMV mutants: (A) NBGP74Km, a double mutant, gO (GP74Km) and GP129 mutant (NRD13); (D) NBNRD13, a GP129 mutant (NRD13); (G) NBGP129FRT/GP74Km, a double mutant, gO (GP74Km) and GP129 (NRD13) plus ectopic expression of full length GP129myc (*GP26/GP26* locus). Virus determined by GFP reporter gene spread across the cell monolayer. Results shown are at approximately 18 days post BAC transfection for both GPL and EPI cells for each virus. Panels B and C, NBGP74Km. Panels E and F, NBNRD13. Panels H and I, NBGP129FRT/GP74Km.

In a previous study, it was demonstrated that GPCMV forms a homolog gH/gL/gO glycoprotein triplex and that gO (GP74) was only essential in lab adapted virus which lacked a PC [36]. In an effort to demonstrate that the PC is more important than the triplex complex for infection of epithelial cells, a series of gO (GP74)/ GP129 mutant GPCMV BACs were transfected onto GPL or epithelial cells to evaluate virus spread. Three GPCMV BAC mutants (Fig 8) were used: (1) GP74Km, which contains a GP74 knockout on a GPCMV BAC that lacked a full length GP129 [36]; (2) NRD13, GPCMV BAC that lacked full length GP129; (3) GP129FRT/GP74Km, GPCMV BAC that encoded a GP74 knockout and a full length GP129 cDNA in the GP25/GP26 intergenic locus. Transfection of GP74Km GPCMV BAC DNA (1) onto GPL or epithelial cells failed to enable the development of viral plaques but instead remained as single transfected cells which could be identified by GFP reporter gene expression (Fig 8A–8C). Transfection of NRD13 (GP129 mutant) GPCMV BAC (2) onto GPL or epithelial cells resulted in the development of viral plaques and spread on fibroblast cells but failed to produce virus on epithelial cells (Fig 8D–8F). Transfection of GPCMV BAC GP129FRT/GP74Km onto GPL and epithelial cells resulted in the development of viral plaques on both cell types (Fig 8G–8I). Importantly, virus derived from BAC GP129FRT/GP74Km demonstrated that gO was not completely essential for epithelial cell infection.as virus spread across the entire monolayer of epithelial cells. Although GPCMV infection of epithelial cells could occur in the absence of gO via the PC, the gB glycoprotein was presumed to be required for epithelial cell infection as demonstrated for fibroblast cells [36]. In an additional experiment, a PC+ /gO+/gB (GP55) negative mutant GPCMV BAC (GP129FRT/GP55Km) when transfected onto epithelial cells failed to produce infectious virus. In contrast, a gB rescue virus had restored epi-tropism which emphasized the essential nature of the gB protein for epithelial infection despite the requirement for a PC (S14 Fig)[36]

Since infection of epithelial cells occurred for a PC+/gO negative virus but not for a PC negative/gO+ virus, we concluded that epithelial cell infection is not absolutely dependent upon gO or the gH/gL/gO triplex but the PC is essential for epithelial cell infection. Admittedly, this particular assay was relatively crude since it fails to fully discriminate between cell to cell spread and infection from cell release virus. However, the strategy demonstrated an importance of the PC for epithelial cell infection. A similar approach defined the essential nature of the GPCMV glycoproteins (gB, gH, gL, gO, gM and gN) for fibroblast cells [36]. In HCMV, a PC+/gO+ virus can more easily infect epithelial cells than a PC+/gO negative virus [67]. Therefore in HCMV, gO has an undefined role in infection of cell types other than fibroblasts via an undefined mechanism [36]. The role of gO in potentially enhancing GPCMV infection of epithelial cells and other cells types remains to be evaluated.

In HCMV, PC dependent infection of epithelial and endothelial cells occurs via an endocytic pathway that requires an acid flux in the endosome [61]. The antibiotic bafilomycin prevents HCMV infection of epi/endothelial cells by inhibiting the ATPase and subsequent acidification of the endosome [61, 97, 98]. Pretreatment of guinea pig epithelial cells with 50nM bafilomycin dramatically inhibited virus infection of epithelial cells but did not greatly impact on fibroblast cell infection (S15 Fig). This potentially indicated that GPCMV entry into epithelial cells is via a similar pathway to HCMV. More focused future studies on the process of GPCMV entry into epithelial cells might provide better insight into the entry pathway for GPCMV and the similarity to HCMV. Analysis of the level of PC compared to gH/gL/gO on GPCMV particles might be worthy of future investigation as this might help to define virus cell tropism.

### Ectopic expression of GP129 and improved pathogenicity in animals

**Fig 9:**
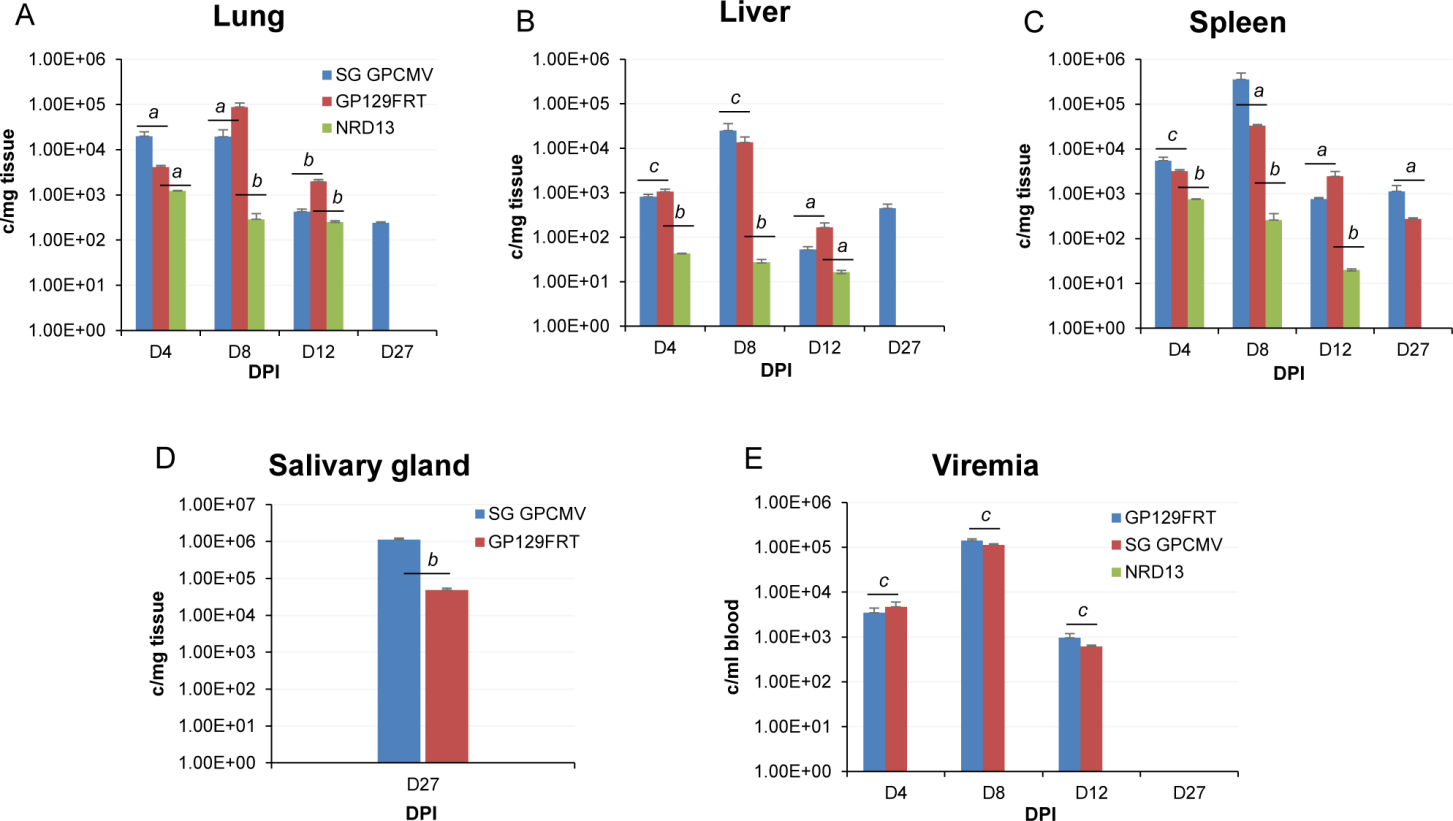
GPCMV pathogenicity studies in seronegative animals. Comparative dissemination of viruses to target organs. Three separate groups (n = 12 per group) of animals were infected with either SG GPCMV, GP129FRT or NRD13 viruses (10^6^ pfu). At various days (4, 8, 12 and 27 days post infect infection, DPI), 3 animals per group were evaluated for viral load in target organs by real time PCR of tissue extracted DNA. Viral load plotted as viral genome copies/mg tissue. Salivary gland tissue was only evaluated at day 27. Graph: **A**, lung; **B**, liver; **C**, spleen; **D**, salivary gland. Blood viremia at 4, 8, 12 and 27 DPI is shown in **E** for viruses GP129FRT and SG GPCMV and plotted as genome copies/ ml blood. Viremia for NRD13 was below the level of detection. Statistical analysis performed by Student t test comparing viral load of GP129FRT vs SG GPCMV and GP129FRT vs NRD. Statistical groups: *a*) p <0.05; *b*) p < 0.005; *c*) NS non-significant.

In an effort to determine if expression of the PC increased virus pathogenicity, comparative animal studies were performed with different GPCMV: GP129FRT (group 1); SG GPCMV (group 2); and NRD13, BAC derived GPCMV (group 3). Seronegative animals were randomly divided into three groups (n = 12 per group) and animals were each inoculated with 10^6^ pfu of virus (either GP129FRT, SG GPCMV or NRD13 dependent on their specific group). At various time points (4, 8, 12 and 27 days post infection), three animals per group were euthanized and the viral load in target tissue and blood were determined by real time PCR as described in materials and methods. The results of the viral pathogenicity study are shown in Fig 9. Statistical analysis (Student t test) was carried out for GP129FRT vs SG GPCMV and GP129FRT vs NRD13 on tissue from similar target organs at similar time points. Overall, the GP129FRT virus had a dissemination pattern that resembled SG GPCMV during the first 12 days of infection in target organs lung, liver and spleen (see Fig 9A–9C). The viral load in the salivary gland was not evaluated until day 27 and was detected for both GP129FRT and SG GPCMV. The viral load in the salivary gland was approximately 1 log higher for the SG GPCMV compared to GP129FRT which was significant (*p* <0.005). Additionally, at day 27 the SG GPCMV could be detected in the target organs lung, liver and spleen, whereas GP129FRT could only be detected in the spleen. Overall, in the target organs there was a statistically significant difference (*p* <0.05 to <0.005) in viral load between GP129FRT and SG GPCMV except in the liver (D4 and D8) and spleen at D4. Both SG GPCMV and GP129FRT exhibited similar viremia levels at days 4, 8 and 12 post infection, with peak levels detected at 8 days post infection. No viremia was detected at day 27.

In contrast to GP129FRT and SG GPCMV, the parental BAC derived NRD13 virus (encoding a truncated GP129) was highly attenuated in the animal model, see Fig 9. NRD13 was detected in lung, liver spleen at days 4, 8 and 12 post infection but at substantially reduced levels compared to GP129FRT and SG GPCMV. At all comparable time points, viral titer in all NRD13 tissues were significantly lower with *p* < 0.005 when compared to GP129FRT (except D4 lung and liver, *p* < 0.05). NRD13 failed to be detected in any target organs at day 27 and was not present in the salivary gland. In contrast to the other viruses, NRD13 viremia at all time points was below the level of detection (Fig 9E). Presumably, the inability of NRD13 to form a pentameric complex and inability to infect a wider range of cell types precluded the ability of NRD13 to effectively disseminate or replicate in target organs. Overall, it was concluded that restoration of an ability to express a missing full length GP129 protein resulted in a virus that could not only infect epithelial cells in tissue culture but also had a greater pathogenicity in the animal model compared to the parental GP129 mutant virus (NRD13). Importantly, the GP129FRT virus was capable of disseminating to the salivary glands unlike the GP129 mutant.

### Ectopic expression of GP129 and increased viral congenital infection

The placenta consists of both epithelial and endothelial cells, the improved tropism of the GP129FRT virus for epithelial cells and other cell types in vivo could also potentially increase the congenital transmission rate of the virus compared to the parental NRD13 virus. Seronegative pregnant dams were challenged with 10^6^ pfu of NRD13 (group 1, n = 8) or GP129FRT (group 2, n = 11) at late second trimester via subcutaneous inoculation and animals were allowed to go to term. The viral load in pup target organs (liver, lung, spleen, brain) of live or still born animals was evaluated. Table 1 has the mortality outcome for live vs still born pups for the groups. Table 2 has the viral load in the target organs of the pups (live and dead).There was a higher number of still born pups in the GP129FRT group (9 pups, 17.3%) compared to the NRD13 group (2 pups, 6.5%).

**Table 1:** Congenital CMV pup birth outcome.

Groups	*n*	Litters					# pups	
		Total	Live only	Dead only	Mixed	Reabsorbed	Live/ dead	% mortality
NRD13	8	7	6	0	1	1	29/2*^a^*	6.5%
GP129FRT	11	10	5	0	5	1	43/9*^a^*	17.3%

^*a*^ *p* = non-significant (Fisher’s exact test)

**Table 2:** Congenital CMV infection viral load in pups and transmission rate.

Groups	CMV+ pups	Lung*^a^*	Liver*^a^*	Spleen*^a^*	Brain*^a^*	Placenta*^a^*
NRD13	1/31 (3.22%)	0 (0/31)	0 (0/31)	0 (0/31)	1.50x10^3^ (1/31)	(0/7)
GP129FRT	20/52 (38.46%)	4.65x10^2^ (15/52)	5.19x10^2^ (10/52)	1.53x10^2^ (12/52)	1.56x10^3^ (10/52)	4.36x10^3^ (2/2)
*P* value	0.0002*^b^*	0.0006*^c^*	0.011*^c^*	0.0028*^c^*	0.047*^c^*	0.028*^c^*

^*a*^Viral load expressed in CMV copies/mg tissue (No. of CMV+/total tissue sample)

^*b*^Transmission rate (GPCMV detected in at least one organ) Fisher’s exact test

^*c*^Comparison of CMV+ samples in each tissue group GP129FRT vs NRD13 (Fisher’s exact test)

Overall, there was a greater incidence of organs positive for virus in the GP129FRT group compared to the NRD13 group (*p* < 0.05). In the latter group, only the brain was detected positive for virus in one pup and all organs were negative in all other animals. In the GP129FRT group, 20/52 pups had virus detected in various organs (brain, liver, spleen and lung). The transmission rate for the GP129FRT virus was 38.46% compared to 3.2% for the NRD13 virus, which was statistically significant (*p* = 0.0002). It should be noted that none of the still born pups in the NRD13 group were positive for virus and their death in utero was likely a complication of pregnancy and not associated with congenital CMV infection.

**Fig 10:**
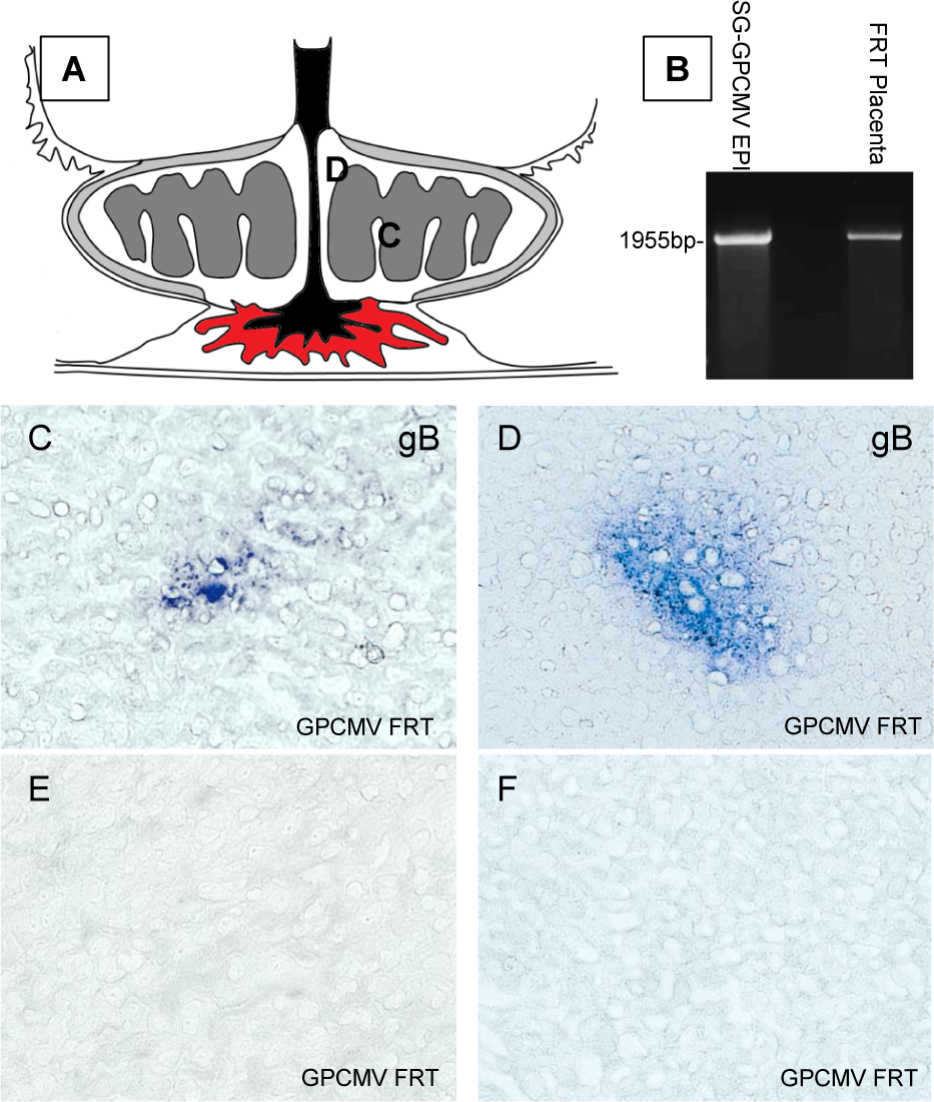
Detection of GP129FRT in guinea pig placenta. Placentas were collected from a euthanized pregnant (3^rd^ trimester) Hartley guinea pig at 22 days post infection with GPCMV129FRT (10^6 pfu subcutaneous injection). Immunohistochemistry was performed on frozen cryostat sections (5 um) as described in materials and methods and stained for GPCMV gB glycoprotein. **A.** Cartoon of the guinea pig placenta (based on ref [20]). C and D indicate the location of the gB antigen detected (labyrinth) in panels C and D. **B.** Agarose gel of the *GP128-133* locus PCR amplified from DNA extracted from EPI cells or placenta tissue with P1 and P2 primers (Fig 1). Left lane SG GPCMV (epi cells) and right lane GP129FRT (placenta). **C** and **D.** Immunohistochemistry stain of placenta tissue for GPCMV gB. **E** and **F.** Control immunochemistry placenta sections (secondary antibody only).

Only a limited number of term placentas were available for evaluation of viral load (2 for GP129FRT group and 7 for NRD13 group) but only the GP129FRT infected animals had CMV positive placentas (see Table 2). The presence of GP129FRT virus in the placenta (3^rd^ trimester) of an additional pregnant guinea pig was evaluated by immunohistochemistry at day 22 post-infection. Fig 10 shows the results for immunohistochemistry staining of cryostat sections of placenta for GPCMV gB antigens. The presence of virus in placenta tissue was also verified by DNA extraction and PCR. Fig 10 Panel A shows a cartoon of the placenta structure and indicated regions where viral antigen was detected in cryostat sections (C and D). Panel B shows an agarose gel PCR analysis for DNA extracted from virus infected epithelial cells or placenta section. The PCR was for the *GP128-GP133* locus and only a full length locus (2 kb) was detected. Panels C and D are sections stained for GPCMV gB protein. Representative control sections (no primary antibody) are shown in panels E and F. Virus was mainly detected in the interface of the labyrinth region of the placenta. These results confirm the presence of viral antigens in the placenta. We concluded that the GPCMV congenital transmission rate was highly dependent upon the virus encoding a functional GP129/ PC. A wider range of virus cell tropism would presumably be a requirement for effective congenital transmission to the fetus since the maternal fetal barrier in the placenta consists of a layer of epithelial syncytiotrophoblast cells [99, 100].

## Discussion

Electron microscopy histopathology studies of guinea pig salivary gland (duct cells) and placental tissue (trophoblast cells) from GPCMV infected animals suggested that GPCMV can infect epithelial cells [21, 101]. Retrospectively, these were important observations in the context of viral tropism and recent identification of a novel mechanism of HCMV infection of epithelial and endothelial cells and other cell types [60, 61, 63, 64]. Our recent knockout mutagenesis studies of GPCMV glycoprotein genes (encoding homologs of gB, gH, gL, gO, gM, or gN) demonstrated a conservation of essential function and homolog glycoprotein complex formation (gB, gH/gL/gO and gM/gN) between HCMV and GPCMV [36, 102]. This present study demonstrated that the *UL128-131* homolog locus (*GP129-GP133*) [28] was necessary for GPCMV infection of guinea epithelial cells in tissue culture and that tropism is dependent upon the ability to form a pentameric homolog complex which is structurally present in the viral particle. GPCMV knockout mutagenesis demonstrated that pentameric complex formation and epithelial tropism was dependent upon an ability of the virus to express wild type GP129, GP131 and GP133 but that GP128 was non-essential for complex formation, nor epithelial tropism. Furthermore, improved epithelial tropism, pathogenicity and congenital infection in the animal model could be established for a mutant virus (*GP129* mutant in the *GP128-133* locus) via ectopic expression of a wild type *GP129* cDNA and subsequent ability to form a homolog pentameric complex. Importantly, GPCMV pentameric complex formation is dispensable for infection of fibroblast cells, as is the case for HCMV. Curiously, Auerbach et al. [90] showed an enhancement of GPCMV fibroblast infection associated with a full length *GP129-133* locus. Our studies with both GPL fibroblast cells and in house primary fetal embryo fibroblasts did not determine a dependence upon an intact *GP128-133* locus for fibroblast virus infection. Similarly, Inoue and colleagues [26] did not see PC dependence for infection of primary or immortalized guinea pig fibroblast cells [28]. Surprisingly, Auerbach and colleagues failed to demonstrate a specific tropism for endothelial cells in virus with an intact *GP128-133* locus compared to virus with a deletion in that locus [90]. Based on our epithelial tropism data and in vivo pathogenicity studies, the prediction would be that PC positive GPCMV would exhibit increased tropism to a wider range of cell types. This increased range would include endothelial cells. HCMV exhibits the same requirement for the PC for cell entry in both epithelial and endothelial cells [63, 64]. In addition to the current reported epithelial cell line, we have recently isolated two guinea pig placenta derived epithelial cell lines which have the same stringency of requirement for the GPCMV pentameric complex [103]. Importantly, studies on guinea pig leukocyte cells suggest that the PC is necessary for infection on these cell types, which is a similar requirement as seen for HCMV [93, 104].

Potentially, in the Auerbach et al. study [90], the endothelial cell population, which were isolated by flow cytometry using cross reacting antibodies, there was a low level of contaminating fibroblast cells, which would prevent a contrasting requirement for the pentameric complex for cellular infection. In our study, epithelial cell isolation was carried out by conventional cloning strategy and antibody staining of cells for cytokeratin marker. Additionally, the immortalized cell lines were re-cloned and characterized (S3 Fig). In contrast, Auerbach et al. [90], elected to employ RT-PCR analysis of total cell monolayer lysate to characterize their primary endothelial cells despite von Willebrand factor antibody being successfully used to identify guinea pig endothelial cells in previous studies [105]. Consequently, the uniformity of their endothelial cell line remains to be fully confirmed. Importantly, newly isolated epithelial cell lines from the guinea pig placenta demonstrate that GPCMV infection of specialized placental cells requires the PC [103] which further supports the hypothesis that increased congenital transmission is PC dependent. This also explains the increased congenital infection rate observed in virus encoding the full length *GP128-133* locus [29, 106].

Transient expression of the homolog components of the penatmeric complex (gH, gL, UL129, UL131, UL133) in epithelial cells resulted in complex formation in the absence of other viral components and this confirmed the results by Auerbach et al. [90] which used purified recombinant baculovirus expressed protein to form pentameric complex in vitro. Importantly, a natural C-terminal deletion mutant of the GP129 protein (codons 102–179) from a virus lacking epithelial tropism (NRD13) was incapable to forming a pentameric complex with other complex components. The importance of C-terminal domain of GP129 for stable PC formation in recombinant GPCMV remains to be further evaluated.

In this current report, PC mutant studies were limited to GP129 but the generation of mutants of other PC components (eg. GP131 or GP133) would be worth evaluation in future studies to aid in the definition of critical domains for complex formation. In HCMV, the UL128 protein interaction with gH/gL is a potentially important key stage because of disulphide bond formation with gL [55]. In our GPCMV studies, we demonstrated that interactions with gL as well as gH is important for PC formation (Fig 2 and S7 Fig). Additionally, gH/gL/GP129 triplex complexes occur (Fig 4), but disulphide bond formation was not investigated. Most certainly, conservation of cysteine amino acids means that the GP129 has the possibility of interacting with gL in the same specific manner. Importantly, the GP129 mutant was unable to form a triplex complex with gH/gL. Triplex complexes with gH/gL and GP131 or GP133 could also be demonstrated. Subcomplex formation has been demonstrated to occur for HCMV between various components of the PC [62]. An evaluation of the stoichiometry of these subcomplexes in various cell types might be important in the determination of their influence on the immune response of the host. The GPCMV PC is highly immunogenic but not individual unique components (GP129, GP131 or GP133) based on newly developed ELISA assays [103]. Consequently, the homolog PC would appear an important target antigen in GPCMV as is the case for the PC in HCMV.

A fundamental question is what dictates formation of a gH/gL/gO triplex, or gH/gL/UL128-131 pentamer in HCMV, and the homolog complexes in GPCMV infected cells. In GPCMV, GP129, GP131 and GP133 would have a theoretical 3:1 advantage over gO for interaction with gH/gL and the same would be the case for HCMV (UL128, UL130 and UL131). In a recent model for HCMV pentamer complex formation, Ciferri et al [55] proposed that UL128 and gO each compete to form a disulphide bond with gL at codon 144 in an exclusive way in the gH/gL complex and that this is the most important interaction since UL130 and UL131 attach to gH/gL via non-covalent bonds. Alternatively, in HCMV an additional protein encoded in the ULb’ region (UL148) has been proposed to regulate gH/gL/gO or gH/gL pentamer formation in an undefined manner [107]. Identification of a homolog UL148 might be worthwhile exploring in future GPCMV studies as would evaluation of the ability of GP129 to bind gH/gL competitively in the presence of gO.

Curiously, gO in human and animal CMV is heavily N-glycosylated. In a previous publication [36], we investigated the importance of the N-glycosylated or non-glycosylated gO protein in GPCMV gH/gL/gO triplex formation. Both wild type (N-glycosylated) gO and a gO mutant (lacking N-glycosylation sites) were equally capable of forming a triplex. Additionally, in GPL cells infected with GPCMV, the wild type gO could be detected as both glycosylated and non-glycosylated in equal amounts but in transient plasmid expression studies only the N-glycosylated gO could be detected [36]. Potentially, the gO glycosylation state could have ramifications for the steady state of gH/gL available to interact with pentameric complex components. A heavily N-glycosylated gO protein may have a more effective interaction with the endoplasmic reticulum (ER) calnexin chaperone protein system [108]. This in turn may enhance the movement of gO or gH/gL/gO complex through the ER resulting in more efficient virion maturation and egress. Consequently, if the glycosylation status of gO in fibroblast vs epithelial cells was different then potentially this might influence the ability of one complex to form over another and subsequently augment viral tropism by the variation of triplex vs pentameric complex on the outside of the viral particle. However, to more fully investigate these possibilities in GPCMV would require the development of a gO specific antibody for full evaluation of these effects.

The animal pathogenicity studies demonstrated that a restoration of an ability of the virus to form a pentameric complex not only resulted in epithelial tropism but also increased viral pathogenicity as well as congenital transmission rate (Fig 9, Tables 1 and 2). The ectopic expression of GP129 cDNA resulted in a virus with similar pathogenicity pattern to SG GPCMV and importantly the virus reached the salivary glands. In contrast, the parental GPCMV BAC derived virus (NRD13) lacked viremia and poorly disseminated. Additionally, the GP129FRT virus had a congenital transmission rate of 38.4% compared to 3.2% for NRD13. Congenital GPCMV challenge studies are normally carried out with virus stock generated by serial passage in the animal and virus generated as salivary gland stock. The SG GPCMV stock of wild type virus has a congenital transmission rate between 55–75% based on previous publications. Potentially, passage of GP129FRT in animals to generate a salivary gland stock might be enhance the congenital rate of the virus and these studies are currently underway.

The basis for the viral attenuation occurring in fibroblast cells (*GP128-133* locus mutation) is undetermined. Most certainly there appears to be a bias in fibroblast cells for the production of cell free virus encoding the triplex compared to virus that also encodes the pentameric complex. The spliced nature of the encoded *GP129* and *GP131* genes might be a contributing factor to a rate limiting step on expression kinetics. Alternatively, the gO glycosylation status in fibroblast cells compared to epithelial or other cell types might influence triplex (gH/gL/gO) formation. Overall, these factors might also be a basis for the rapid generation of *GP128-133* locus mutants when SG GPCMV is serially passaged on fibroblast cells. However, this remains to be further investigated and may potentially require the development of additional epithelial and fibroblast cell lines to demonstrate that this phenomenon is not limited to the cell lines used in this study.

In summary, this study demonstrated that the *UL128-131* homolog locus (*GP129-GP133*) is necessary for GPCMV to form a homolog PC. Additionally, loss by GPCMV of the capability to form the PC impaired epithelial cell tropism and attenuated the virus in the animal model. Importantly, restoration of GPCMV epithelial tropism resulted in increased pathogenicity and congenital transmission rates. Overall, these findings strengthen the guinea pig as a highly relevant model for HCMV congenital infection and the development of CMV vaccine or intervention strategies against congenital infection. Additionally, these studies emphasize the importance of the PC for congenital transmission and strongly suggest that a vaccine aimed at preventing congenital infection should incorporate the pentameric complex as part of a vaccine design.

## Materials and Methods

### Cells, viruses and oligonucleotides

GPCMV (strain 22122, ATCC VR682), first and second generation GPCMV BAC [26, 27] derived viruses were propagated on guinea pig fibroblast lung cells (GPL; ATCC CCL 158) in F-12 medium supplemented with 10% fetal calf serum (FCS, Life Technologies), 10,000 IU of penicillin/liter, 10 mg of streptomycin/liter (Life Technologies), and 7.5% NaHCO_3_ (Life Technologies) at 37°C/5% CO_2_. Virus titrations were carried out on six-well plates. Plaques were stained with 10% Giemsa stain or visualized by fluorescence microscopy. High titer stock viruses were generated as previously described [36]. Sucrose gradient purified stocks were initially generated as high titer stock virus and subsequently purified on a sucrose gradient following a previously described protocol [109]. Guinea pig epithelial cells were initially isolated as primary renal epithelial tubule cells from the kidney of a Hartley guinea pig by conventional clonal ring procedure. Primary epithelial cells were initially maintained on collagen (Life Technologies) coated plates in epithelial cell growth media (Applied Technology) supplemented with 10% FCS (Life Technologies) and antibiotics as described for F-12 media. Transformed epithelial cells were maintained in high glucose DMEM with sodium pyruvate (Life Technologies) supplemented with 5% FCS (Life Technologies) and antibiotics as described for F-12 media. Epithelial cells were maintained at 37°C/5% CO_2_ and characterized as epithelial cells by characteristic cobbled stone appearance of the cell monolayer as well as staining positive for cytokeratin marker (anti-pancytokeratin antibody and anti-cytokeratin 18 antibody, Cell Signaling). Tissue culture stocks of GP129FRT and SG GPCMV were generated on epithelial cells. All oligonucleotides were synthesized by Sigma-Genosys (The Woodlands, TX) and are listed in S1 Table.

### Transformed guinea pig renal epithelial cells

Primary Hartley guinea pig renal epithelial cells were isolated following protocol by [110]. Subsequently, cells were transformed by transduction with defective lentiviruses encoding SV40 TAg or HPV E6/E7 (Applied Biological Materials Inc.) following manufacturer’s protocol. Cells were re-seeded onto collagen coated 100mm dishes and propagated in high glucose DMEM with sodium pyruvate (Invitrogen) supplemented with 10% fetal calf serum and 1X antibiotic-antimycotic (Life Technologies) and 37°C/5% CO_2_. Rapidly growing colonies were isolated by clonal ring procedure and subcloned onto fresh monolayers of collagen coated plates and maintained as separate cell lines and aliquots of cells in cryopreserve media (Gemini Bioproducts) and stored in liquid nitrogen. Transformed cells were verified as epithelial by positive staining for cytokeratin marker by western blot or immunofluorescent staining using anti-pancytokeratin (Cell Signaling Technology) and anti-cytokeratin 19 (Applied Technology) using a previously described protocol [36]. SV40 T antigen transformed cells were poorly supportive of GPCMV infection and were not used in the reported studies. HPV E6/E7 transformed cells were supportive of GPCMV infection and used extensively in the studies.

### Ethics

Guinea pig (Hartley) animal studies were carried out under approval by IACUC at Texas A&M University, permit 2013#013. All study procedures were carried out in strict accordance with the recommendations in the “Guide for the Care and Use of Laboratory Animals of the National Institutes of Health.” Animals were observed daily by trained animal care staff, and animals requiring care were referred to the attending veterinarian for immediate care or euthanasia. Terminal euthanasia was carried out by lethal CO_2_ overdose followed by cervical dislocation in accordance with IACUC protocol and NIH guidelines. Animals purchased from Charles River Laboratories were verified as seronegative for GPCMV by toe nail clip bleed and anti-GPCMV ELISA of sera as previously described [36].

#### Pathogenicity studies

Seronegative animals were randomly divided into three groups (n = 12 per group): GP129FRT (group 1); SG GPCMV (group 2); and NRD13, BAC derived GPCMV (group 3). Animals were each inoculated with 10^6^ pfu of virus via subcutaneous injection (either GP129FRT, SG GPCMV or NRD13, dependent on their specific group). At various time points (4, 8, 12 and 27 days post infection), two animals per group were euthanized and the viral load in target tissue (liver, lung, spleen, salivary glands) and blood were determined by real time PCR.

#### Congenital GPCMV study

Seronegative pregnant dams were challenged with 10^6^ pfu of NRD13 (group 1, n = 8) or GP129FRT (group 2, n = 11) at late second trimester via subcutaneous inoculation and animals were allowed to go to term. The viral load in target organs (liver, lung, spleen, brain) of live or still born pups was evaluated by real time PCR. Additionally, for immunohistochemistry studies of infected placenta, a single pregnant dam was challenged with GP129FRT as described above. At 22 days post infection, the dam was euthanized and placenta recovered for evaluation of virus infection via immunohistochemical staining. Frozen placenta was sectioned using a cryostat (5 um thickness). Sections were fixed and analyzed for the presence of GPCMV using mouse anti-gB antibody (29:29) and anti-mouse IgG elite ABC kit (Vectastain) following manufacturer’s protocol.

### Real time PCR

Blood and tissues (lung, liver, spleen) were collected from euthanized guinea pigs to determine the viral load. For pups from congenital infection studies, blood and tissues (lung, liver, spleen, brain) were collected within 3 days post birth. Pup specific placenta was collected and preserved for DNA extraction when applicable. For tissue DNA extraction, FastPrep 24 (MP Biomedical) was used to homogenize tissues as a 20% weight/volume homogenate in Lysing Matrix D (MP Biomedicals). To obtain DNA from whole blood, 500ul of blood was collected (by toe nail clip bleed) into tubes containing ACD anticoagulant and 200μl of blood was subsequently used per extraction. DNA was extracted using the QIAxtractor (Qiagen) according to manufacturer’s liquid (blood) or tissue protocol instructions. Viral load was determined by real time PCR on Lightcycler 480 (Roche Applied Science). Primers and hydrolysis probe were designed using the Lightcycler Probe Design2 program to amplify a product from the GPCMV GP44 gene: Forward primer 5’TCTCCACGGTGAAAGAGTTGT; Reverse primer 5’GTGCTGTCGGACCACGATA; hydrolysis probe 5’FAM-TCTTGCTCTGCAGGTGGACGA-BHQ1. PCR master mix contained Lightcycler Probes Master (Roche Life Science), 0.4 μM primers and 0.1 μM probe, 0.4U uracil N-glycosylase (UNG) in 25μl total reaction volume including 10 μl of DNA per reaction. Standard controls and no template controls (NTC) were run with each assay for quantification. Lightcycler480 amplification parameters were: UNG step for 10 minutes at 40°C followed by activation at 95°C for 10 minutes, then 45 cycles of denaturation at 95°C for 15s, annealing at 56°C for 15s, elongation at 72°C for 10s. Data was collected by ‘single’ acquisition during the extension step. Standard curve was generated using GPCMV GP44 plasmid [33] for quantification and assay sensitivity. The sensitivity of the assay was determined to be 5 copies /reaction. Viral load was expressed as copy number/ml of blood or copy number/mg tissue. Results calculated were a mean value of triplicate PCR runs per sample.

### Cloning of GPCMV genes and generation of knockout shuttle vectors

The predicted GPCMV coding sequences were based on the complete 22122 viral genome sequence (Genbank accession #AB592928.1). The specific gene coding sequence co-ordinates are: *GP74*, gO, (117,992–119,104); *GP75*, gH, (119,553–121,724); *GP115*, gL, (180,216–180,992); *GP128* (195,713–196,768); *GP129* complement (196,745–197,003; 197,081–197,206; 197,285–197,439); *GP131* complement (197,444–197,780; 197,861–198,102); *GP133* complement (198,102–198485) and *GP130* (196,968–197360). Generation of individual shuttle vectors for specific gene knockout (or intergenic insertion of GP129) and construction of transient expression vectors are described in more detail below.

#### GP128-GP133 locus knockout shuttle vectors

In order to carry out a systematic knockout of the GPCMV genes (*GP129*, *GP131* and *GP133*), synthetic gene sequences were generated with unique flanking homologous regions for recombination with the GPCMV genome sequence that also introduced targeted deletion of the majority of the specific gene of interest. Two synthetic shuttle vectors pSYDGP129/131 (GPCMV flanking sequence 197,041–197,291; 198,091–198,300) and pSYDGP133 (GPCMV flanking sequence 198,178–198,360; 198,490–198720) were generated (DNA2.0) to knockout GP129 and GP131 or GP133 respectively. pSYDGP129/131 targeted deletion of GPCMV nucleotides 197,292–198090 (798 base deletion). pSYDGP133 targeted deletion of GPCMV nucleotides 198,361–198,489 (138 base deletion). The shuttle vectors were engineered to carry a *Bam*H I site in the deleted locus. Subsequently the shuttle vectors were modified by insertion of a kanamycin/FRT cassette [36] into the unique BamHI site between left and right flanking arms of the respective shuttle vectors to generate pSYDGP129/131KmFRT and pSYDGP133KmFRT. For *GP128* knockout, the complete coding sequence was PCR amplified as a BamHI fragment with the primer set GP128F and GP128R (S1 table) and cloned into pUC19 to generate pUCGP128Bm. A kanamycin/FRT cassette with flanking *Eco*R V sites was cloned into a unique *Eco*R V site within the GP128 coding sequence to disrupt the ORF at codon 176 (pGP128KmFRTEcV). S1 Fig shows the location of the GPCMV sequence modifications in the *GP128-GP133* locus.

#### GP25/GP26 locus shuttle vector

For ectopic expression of GP129myc, the ORF was placed under SV40 promoter control, a shuttle vector was generated that introduced a cassette into an intergenic locus between *GP25* and *GP26*. This locus was selected as both genes co-terminate in this locus from opposite directions and insertion into this intergenic locus has previously been shown not to impact on the virus growth kinetics [25]. First the *GP25/GP26* locus (GPCMV (strain 22122) 37,672–38,953bases) was PCR amplified as an *Eco*R I to *Hind* III fragment partially encoding *GP25* and *GP26* using primers FGP25 and RGP26 (S1 Table) and cloned into pUC19 as an *Eco*R I/ *Hind* III fragment to generate pUCGP25/GP26 (S11 Fig). Next, the SV40 promoter/ SV40 polyA sequence from pSI (Promega) was isolated as a *Bgl* II/ *Bam*H I fragment and cloned into the unique *Bam*H I site (GPCMV 22122 strain, position 38,538 bases) in the intergenic locus between *GP25* (sense strand) and *GP26* (complementary strand) genes in linearized pUCGP25/GP26 to generate pSIGP25/GP26. A synthetic intron was removed from the shuttle vector by *Pst* I/*Sal* I collapse of the polylinker and insertion of a 50 bp linker sequence (Link1, see S1 Table) to generate pGP25/26Link1 which now carried unique *Bgl* II and *Bam*H I sites. A *Bam*H I fragment of GP129myc was cloned into *Bgl* II cut pGP2526Link1 to generate pGP129Link1. Subsequently, a *Bam*H I Km cassette [36] flanked by FRT sites was cloned into pGP129link cut with *Bam*H I to generate pGP129limkKmFRT which was the final recombination shuttle vector expressing GP129. The nucleotide sequence of the *GP25/GP26* locus and the cloning strategy for generation of the recombination shuttle vector and locus modifications are shown in S10 Fig and S11 Fig.

#### Construction of GPCMV gH, gL and GP128 tagged mammalian expression vectors

The gH (*GP75*), gL (*GP115*) and gO (*GP74*) ORFs were additionally separately cloned into expression vectors that also tagged the C-terminal domain for easy detection of the recombinant protein in transfected cells. For GFP tagged gH, the *GP75* ORF (missing the stop codon) was PCR amplified as a *Bam*H I fragment using primers FgHBm and RgHBmNostop (S1 Table) and cloned inframe into GFP fusion expression vector pAcGFP-N1 (Clontech) cut with *Bam*H I. This introduced a GFP tag in-frame into the C-terminal domain of the gH ORF and placed the *GP75* under a HCMV MIE promoter control. This modified plasmid was designated pAcGFPNgH. For mCherry tagged gL, the *GP115* ORF (missing the stop codon) was PCR amplified as a *Hind* III fragment using primers FgLHd and RgLHdNostop (S1 Table) and cloned inframe into mCherry fusion expression vector pmCherry-N1 (Clontech) cut with *Hind* III. This introduced a mCherry tag in-frame into the C-terminal domain of the gL ORF and placed the *GP115* under a HCMV MIE promoter control. This modified plasmid was designated pmCherryNgL. A GP128 FLAG tagged vector was generated by PCR using the primer set GP128F and GP128R (S1 Table) cloned as a *Bam*H I fragment into pCMV3Tag (Agilent Technology). The GP128 ORF PCR lacked a stop codon and the *Bam*H I site enabled inframe cloning that introduced a 3xFLAG tag into the C-terminus of GP128 (pFLAGGP128).

#### GP129, GP130, GP131 and GP133 tagged mammalian expression vectors

C-terminal epitope tagged versions of GP129 (3xmyc), GP131 (3xHA), GP133 (3xFLAG) and GP130 (3xFLAG) cDNAs were generated as synthetic genes (DNA2.0 Inc.) based on the defined coding sequence GenBank: KC503762.1 AB592928.1 and cloned under HCMV MIE promoter control in a high copy plasmid vector (pJ603, DNA2.0 Inc).

#### GP129 C-terminal mutants

A synthetic GP129 ORF was generated for the natural truncated C-terminal mutant NRD13 (codon 102–179 deleted). A HCMV chimeric GP129/UL128 C-terminal mutant was also generated that encoded the terminal 48 codons of HCMV (Merlin strain) UL128 in place of codons 128–179 of GP129. Both GP129 mutants were myc tagged.

### Generation of gene knockout GPCMV BACmids and analysis of GPCMV BAC mutants

An inducible ET recombination system (GeneBridges) was introduced into DH10B bacterial cells containing a second generation GPCMV BAC plasmid [26, 27] using a protocol previously described [32]. Individual GPCMV gene knockout targeting shuttle vectors were linearized with a unique restriction enzyme cutting outside the target gene flanking sequence. Linearized DNA plasmids or PCR products were band isolated and concentrations of DNA were modified to introduce 1μg of linear DNA into each transformation reaction via electroporation [32]. Recombinant bacterial colonies of GPCMV BAC mutants were isolated by chloramphenicol (12.5 μg/ml) and kanamycin (20 μg/ml) antibiotic selection in LB agar bacterial Petri dishes. Bacterial plates were initially incubated at 39°C to remove the ts ET recombination plasmid (Genebridges). Mutant GPCMV BAC DNA was purified by maxiprep kit (Qiagen) and analyzed by separate *Eco*R I and *Hind* III restriction digestions to verify the accuracy of the predicted genome configuration after mutation [26, 27]. Insertion of the kanamycin (Km) drug resistance cassette into the viral genome introduced a novel *Hind* III restriction enzyme site at the site of mutation to enable verification of locus modification. Specific gene modifications were confirmed by comparative PCR analysis between wild type and mutant GPCMV BACs using common flanking primers. The gene knockout for mutants was further verified by sequencing of the PCR product. In order to enable a second round of GPCMV BAC mutagenesis, the original Km cassette inserted into the genome was removed by FLP recombinase strategy if the Km cassette was flanked by FRT sites. The FLP recombination was accomplished by transforming the BAC positive bacteria with a FLP expression suicide plasmid (p707, GeneBridges) (permissive conditions with tetracycline (3ug/ml) at 31°C) and recombinase induction and excision of the FRTKm cassette accomplished following manufacturer’s protocol. A second round of recombination could then be carried out on the GPCMV BAC as described above.

### GPCMV BAC mutagenesis and characterization of results

GPCMV genes *GP128*, *GP129*, *GP131* and *GP133* were individually knocked out by targeted mutagenesis of the GPCMV BAC in bacteria using shuttle vectors carrying a Km drug resistance marker to disrupt each ORF. Targeted recombination knockout of GPCMV genes was performed in the second generation GPCMV BAC [27]. Fig 1 shows the layout of the *GP128-133* locus. S1 Fig shows the annotated nucleotide sequence of the *GP128-133* locus with the location of the genes shaded and the specific deletions introduced for *GP129-GP131* or *GP133* mutants indicated. The *GP129/GP131* mutant was generated with a BamHI FRT Km insertion between flanking sequence on the synthetic deletion shuttle pSYDGP129/131, which deleted GPCMV nucleotides 197,292–198,090 (798 bp deletion within the GP129-GP131 coding sequence, see S1 Fig). The *GP133* mutant was generated by a *Bam*H I FRT Km insertion between flanking sequence on the synthetic deletion shuttle vector pSYDGP133, which deleted GPCMV nucleotides 198,361–198,489 (138 base deletion which included the *GP133* start codon (S1 Fig). The *GP128* gene was modified by insertion of a *Eco*R V FRT Km cassette at a unique *Eco*R V site (GPCMV nucleotide 196,234) in the *GP128* gene of the *GP128* shuttle vector (pGP128KmFRTEcV), which disrupted the GP128 ORF at codon 176. The mutant GPCMV BACs were analyzed by restriction enzyme profile analysis as previously described [36]. Insertion of the Km drug resistance cassette into the viral genome introduced a novel *Hind* III restriction enzyme site at the site of mutation to enable verification of locus modification. Modified GPCMV genomes were analyzed separately by *Eco*R I and *Hind* III restriction enzyme profile analysis. In an effort to limit redundancy the profiles shown for each mutant are either *Hind* III or *EcoR* I analysis. Additionally, two clonal mutants were generated for each knockout but only one is described. Comparative restriction fragment profiles of wild type and mutant GPCMV BAC genomes correctly demonstrated specific sub-genomic fragment modification for all mutants. Designated GPCMV restriction fragment band nomenclature described by Gao and Isom [111] was used to identify specific band shifts, except that the 5’ and 3’ genome terminal ends were considered linked in a covalent closed circle [26]. S12 Fig shows the *GP128* mutant *Eco*R I profile and *Hind* III profiles for *GP129-13*1 and *GP133* mutants compared to the wt GPCMV BAC profiles. Specific gene locus modifications were further verified by PCR analysis and sequencing as previously described [36]. The *GP128* gene encoded in the 4.9 kb *Eco*R I GPCMV genomic fragment (192,215–197,167) was modified in the *GP128* mutant by insertion of the 1.1 kb Km cassette which shifted the fragment to 6 kb (S12 Fig). The *GP129*, *GP131* and *GP133* genes are encoded in the 19.7 kb *Hind* III GPCMV genomic fragment and targeted knockout of these genes introduced a new *Hind* III site encoded in the inserted Km marker. The GP129-GP131 deletion mutant *Hind* III fragment was modified from 19.7 kb to 5 kb and approximately 15 kb (S12 Fig). In the *GP133* deletion mutant the 19.7 kb *Hind* III GPCMV genomic fragment was modified to 6 kb and 14.9 kb fragments (S12 Fig).

Mutant GPCMV BACs were independently subject to an additional round of mutagenesis to introduce a wild type *GP129* (myc tagged) cDNA into the *GP25/26* intergenic locus under SV40 promoter control as described in an earlier section for ectopic expression of GP129. This required excision of the Km FRT cassette from the originally mutated locus by Flp recombinase as described (see above section). Excision of the Km cassette was confirmed by patching of colonies for the loss antibiotic resistance and the integrity of the GPCMV BAC confirmed by restriction profile analysis (S12 Fig). GPCMV BACs retained a FRT site at the original site of excision. *E*.*coli* (DH10B) cells carrying respective GPCMV BAC mutants underwent ET recombination induction and targeted modification with the shuttle vector pGP129limkKmFRT as previously described. *GP25/GP26* locus mutants (carrying GP129myc cDNA) were isolated by Km marker insertion as previously described. Full length GPCMV BAC clones were identified by *Eco*R I restriction profile analysis (S12 Fig) and subsequently confirmed by PCR of the *GP25/GP26* locus and sequencing (S11 Fig). Mutants carrying *GP129* cDNA in the *GP25/GP26* locus were designated: (1) GP129FRT (wt GPCMV BAC); (2) GP128FRT/GP129Link (*GP128* mutant); (3) GP129-GP131FRT/ GP129Link (*GP129-GP131* deletion mutant); (4) GP133FRT/GP129Link (*GP133* deletion mutant). In regard to the ectopic insertion of a GP129 cDNA into the *GP25/GP26* intergenic locus, the modification generated a characteristic altered GPCMV *Eco*R I profile. The 5.2 kb GPCMV *Eco*R I genomic fragment (nucleotides 35,537–40,739) containing the *GP25/GP26* locus was modified by the insertion of the SV40 promoter /GP129 expression cassette/SV40 polyA sequence and Km marker into the *Bam* H I site (nucleotide 38,538). The modified sequence also introduced two novel *Eco*R I sites. As predicted the original 5.2 kb genomic fragment was modified to two novel fragments (3.5 and 3.9 kb). S12 Fig shows the modified profiles for wt GPCMV, *GP128* and *GP129-131* mutants (GP128FRT/GP129Link and GP129-GP131FRT/ GP129Link respectively) but not the GP133 mutant to limit redundancy. An additional GPCMV BAC mutant was engineered into GP129FRT GPCMV BAC which introduced a Km cassette into the *GP74* gene (glycoprotein gO) as previously described [36]. This generated a GPCMV BAC mutant that encoded a GP129 cDNA in the *GP25/26* locus (Km replaced by FRT sites only) and a *GP74* knockout by insertion of a Km cassette which disrupted the ORF at codon 110 [36] (double mutant was designated GP129FRT/GP74Km). The *GP74* mutation was confirmed by *Eco*R I profile analysis of the GPCMV BAC genome. The original 18 kb *Eco*R I genomic fragment (nucleotides 102,796–120,821) that encoded the *GP74* locus was increased in size by 1.1 kb by the insertion of a Km cassette which modified the size of the fragment to 19.1 kb, see S12 Fig.

The gB (*GP55*) knockout mutant was generated on the backdrop of GP129FRT GPCMV BAC. The GP55 gene (94,164–96,869) was knocked out by insertion of a Km cassette which disrupted the ORF at codon 528 as previously described [36]. The insertion of the Km cassette modified the size of the 4470 *Eco*R I subgenomic fragment (91,190–95,659) by approximately 1 kb generating a novel *Eco*R I restriction fragment of approximately 5.5 kb on the GP129FRT/GP55km GPCMV BAC *Eco*R I profile. The positions of the wild type and mutant EcoR I restriction fragments are indicated in S12(vii) Fig.

### Generation of mutant GPCMV

For generation of recombinant viruses, large-scale GPCMV BAC DNA was purified from *E*. *coli* DH10B strain using a maxi plasmid kit (Qiagen). BAC DNA was transfected onto GPL cells in six well dishes using Lipofectamine 2000 (Invitrogen) as previously described [33]. GPCMV BAC transfections were carried out with two independent clones for each gene knockout. Transfections were followed for at least 3–4 weeks for the production of viral plaques. GFP positive viral plaques were detected via microscopy [33]. Non-infectious mutants produced only single GFP positive cells that did not progress to viral plaques. GPCMV mutant BAC transfections were carried out multiple times (minimum of 6 times) for each clone. Excision of the BAC plasmid from recombinant viral genome was carried out by co-transfection of BAC DNA with plasmid encoding CRE recombinase (pCRE), a generous gift from Dr. Mike McVoy (Virginia Commonwealth University). Cre BAC excised virus also lost the GFP reporter cassette encoded on the BAC plasmid and therefore GFP negative plaques confirmed successful BAC plasmid excision. Large scale virus stocks were generated as previously described [33] and additionally epitropic viruses were generated as virus stocks on epithelial cells following the same procedure. A gB knockout (GP129FRT/GP55km) rescue virus was generated by co-transfection of GP129FRT/GP55km BAC with GP55 rescue plasmid as previously described [36].

### Recombinant adenovirus vectors

Recombinant defective adenoviruses (serotype 5) encoding individual epitope tagged components of the pentameric complex were generated as high titer stocks by Welgen Inc. on HEK293 cells. The C-terminal epitopes tagged ORFs from plasmids pAcGFPNgH, pmCherryNgL, pGP129myc, pGP131HA, pGP133FLAG were each placed under HCMV MIE enhancer promoter control in the E1 locus of the defective Ad vectors using a E1 shuttle vector (Welgen Inc.) to generate recombinant defective adenoviruses designated AdgHGFP, AdgLmCherry, AdGP129myc, AdGP131HA, AdGP133FLAG respectively. A defective Ad vector encoding GFP (AdGFP) was also used in control expression studies [36].

### RT-PCR

Time point samples were taken from wild type GPCMV infected GPL cells in a six well dish (moi = 1 pfu/cell) at 48 hr post infection. RT-PCR was performed essentially as described in Coleman et al. [36]. Based on the original analysis of the full length GP128-133 locus [28, 87], there are five genes designated *GP128*, *GP129*, *GP130*, *GP131* and *GP133*. Based on co-linearity to HCMV as well as encoded proteins, two of these genes are direct homologs of HCMV *UL128* (*GP129*) and *UL130* (*GP131*) [28]. *GP133* has a weak homology to *UL131*[90]. Gene expression in the *GP128-133* locus at late stage infection of SG GPCMV was investigated via RT-PCR assay as previously described [36] using the following primer pairs: RTGP128F/RTGP128R; RTGP129F/RTGP129R; RTGP130F/RTGP130R; RTGP131F/RTGP131R; RTGP133F/RTGP133R and control *GAPDH* (GAPDHRTF/GAPDHRTR) as described in S1 Table. Results demonstrated that the previously identified genes were all expressed in SG GPCMV infected cells (S2 Fig).

### Immunoprecipitation assays

Immunoprecipitation (IP) assays were carried out on plasmid transfected or recombinant Ad transduced fibroblast cells using commercial GFP-trap reagent (ChromoTek) or RFP-trap (ChromoTek) following manufacturer’s protocols and inclusion of protease inhibitor cocktail (Pierce) in cell lysates. Samples were subsequently analyzed by SDS-PAGE (4–20% gradient gel) and western blot using specific anti-epitope tag antibodies: HA (Novus Biologicals); FLAG (Novus Biological); GFP (Santa Cruz Biotechnology); Myc-c (Novus Biologicals); and mCherry (Clontech Laboratories). Appropriate secondary anti-mouse or anti-rabbit HRP conjugate (Cell Signaling Technology) were also used following standard western blot protocol as previously described [36].

### Specific GPCMV protein antibodies

Custom antibodies to GPCMV gH, IE2 and GP131 were generated by Genescript to specific peptide sequences (rabbit polyclonal) or to purified recombinant (*E*.*coli*) protein (mouse monoclonal). Rabbit polyclonal antibody sera were separately generated against gH and IE2 using the peptide immunogen of RTDLSSPTEELTSP (for gH) or CRKTRPAKRPRSNDE (for IE2). Mouse monoclonal antibody to GP131 was generated against recombinant purified protein and hybridoma antibody screened for activity to GP131. Rabbit or mouse IgG was column purified and resuspended at a concentration of 100 μg/ml. Antibody specificity was verified by both western blot and immunofluorescence assay of transiently expressed proteins (gH, IE2 or GP131) in fibroblast/epithelial cells using appropriate plasmid expression vectors for gH, IE2 or GP131 following standard protocols [36].

### Bafilomycin inhibition of virus entry by pH-dependent endocytosis

Epithelial cells or GPL fibroblast cells on coverslips six well dishes were pretreated with complete media containing either 0 or 50nM bafilomycin A1 (Sigma) for 1 hr at 37˚C followed by virus infection using FRTGP129 virus (MOI = 1pfu/cell) for 1hr at 37 ˚C. All further incubations were performed at the same concentration as pretreatment in complete media. Cells were fixed at 24 hr post infection in 100% methanol at -20˚C and immunostained for IE2 protein and cytokeratin as described in materials and methods. Counts were made of IE2 positive cells in random fields. Statistical analysis was performed using student T-test on the percent of cells infected in thirty random fields of view, each contained ~100 cell nuclei, for each condition. The number of treated cells infected was represented as a percentage of the number of infected untreated cells. Results shown in S15 Fig.

### Statistical analysis

In pathogenicity studies, viral load in similar organ tissue from GPCMV infected animals at specific days post infection were compared by Student t-test (GP129FRT vs SG GPCMV and GP129FRT vs NRD13). In congenital studies, pup outcome and transmission rates were compared by Fisher’s exact test. GPCMV viral load in specific target organs of pups were compared by Student t-test. All comparisons were two-tailed. Student t-test analysis was also carried out for virus infection of cells in bafilomycin virus cell entry studies (S15 Fig).

## Supporting Information

S1 Table Oligonucleotides used in PCR and RT-PCR reactions. (DOCX)

S2 Table Predicted molecular weights of pentameric complex proteins. (DOCX)

S1 Fig Annotated genome sequence for the *GP128-GP133* locus in SG and ATCC lab adapted deletion virus. GPCMV *GP128-134* sequences based on GenBank Accession # AB592928.1. GPCMV nucleotide base co-ordinates indicated in brackets. *GP128* (195,713–196,768) highlighted in blue; *GP129* (196,745–197,439 complement) highlighted in green; *GP131* (197,444–198,102 complement) highlighted in orange; *GP133* (198,102–198,485 complement) highlighted in yellow *GP134* (198,579–199,262 complement) highlighted in grey. Primers P1/P2 (S1 Table) used for verification of intact or deleted **GP129-133** region are bolded in black. Deleted region (1.6 kb) of lab-adapted virus is underlined (196,925–198,573). Sequence deletion for GP129-GP131 mutant (197,292–198,090) start and end sequence of deletion designated by square (□). Sequence deletion for GP133 mutant (198,361–198,489) start and end sequence designated by circle (**○)**. For the *GP128* mutant, an *Eco*R V site (196,234–196,239) within *GP128* was the site of a Km cassette insertion (**GATATC**) which disrupted the ORF.
(TIF)

S2 Fig RT-PCR assay of genes transcribed in the GP128-133 locus of SG GPCMV. Individual RT-PCR primer sets (see material and methods and S1 Table) were designed for GP128, GP129, GP130, GP131and GP133 based on defined sequences. RT-PCR was performed as previously described [36]. RT-PCR products were analysed by agarose gel electrophoresis. Lanes: 1–3, GP128; 4–6 GP129; 8–10 GP130; 11–13, GP131. Lanes 1, 4, 8, 11 and 16 SG GPCMV infected cells. Lanes 2, 5, 9, 12 and 17 mock infected cells (control). Lanes 3, 5, 9, 12 and 18 no RNA control. Lanes 7,14 and 15,100 bp ladder (NEB).
(TIF)

S3 Fig Characterization of guinea pig epithelial cells. **(i).** GPL fibroblast vs Epithelial cell western blot for cytokeratin 18. Cell lysates from ~1x10^6^ GPL and EPI cells were analyzed by western blot using a 4–20% SDS-PAGE gel and probed with anti-Keratin 18 (DC10) Mouse mAb (Cell Signaling) and secondary anti-mouse IgG-HRP conjugate. **(ii).** Immunofluorescence for cytokeratin expression in EPI cells. Monolayers of EPI (images A, B & C) and GPL (images D, E & F) cells were immunostained using Pan-Keratin (C11) mouse mAb (Images A and D) as described in materials and methods. Cell were also stained with high-affinity F-actin probe, anti-phalloidin-Alexa Fluor 568 (ThermoFisher scientific) (Images B and E). Cells were counterstained with DAPI (merged images C and F). Images were taken at 40X using a Olympus IX81 confocal microscope.
(TIF)

S4 Fig PP ATCC mutant virus is impaired for growth on epithelial cells. EPI and GPL cells were infected at a moi of 1 pfu/cell. At 48 hr post infection cells were fixed and stained for viral (IE2) and epithelial cell markers as described in materials and methods. Immunofluorescence images of EPI cells: A, IE2; B, pan-keratin; C, merged A and B with DAPI stain. Immunofluorescence images of GPL cells: D, IE2; E, pan-keratin; F, merged E and F with DAPI stain. Images at x60 maginification.
(TIF)

S5 Fig Recombinant adenoviruses encoding PC components and co-expression on EPI cells. **(i)** Components of the pentameric complex (gH, gL. GP129, GP131 and GP133) were individually cloned as C-terminal epitope tagged ORFs into recombinant defective adenovirus shuttle vectors and recombinant viruses generated for each component. Genes were expressed under HCMV MIE enhancer expression as illustrated. Predicted encoded protein is indicated for each construct. (ii) Cellular co-localization of pentameric complex components in guinea pig epithelial cells in the absence of other GPCMV proteins. EPI cells were transduced with defective recombinant Ad constructs of the pentameric complex as described in materials and methods. gH expression detected under fluorescence (gHGFP). gHGFP localization (panels A, E, and I), gL expression detected under fluorescence (gLmCherry). gLmCherry localization (panels B, F, & J). GP129myc localization using immunofluorescence anti–myc antibody/ Cy5 (Panel C). GP131HA localization using anti-HA antibody/Cy5 (G). GP133FLAG localization using anti-FLAG antibody/Cy5 (K). Panel D (merged A, B & C) for gH, gL and GP129, Panel H (merged E, F & G) for gH, gL and GP131. Panel L (merged I, J & K) for gH, gL and GP133. Merged images also counterstained with DAPI.
(TIF)

S6 Fig Tunicamycin treatment and expression of GP129, GP131 and GP133 proteins. Transient expression of GP129, GP131 and GP133 was evaluated in the presence or absence of tunicamycin treatment. Separate 6 well plates of epithelial cells were transduced with recombinant Ad vectors encoding GP129, GP131 or GP133. Expression occurred in the presence or absence of tunicamycin as previously described (36). After overnight expression, monolayers were either harvested for western blot analysis (A-C) or fixed for immunofluorescence assay (D-I). Western blot assays (A-C). Lanes: 1, 4 and 7 mock infected cells; 2 and 3 AdGP129myc transduced cells; 5 and 6 AdGP131HA; 8 and 9 AdGP133FLAG. Lanes 3, 6 and 9 represent cells treated with tunicamycin. Immunofluorescence assays: D and G, AdGP129myc; E and H, AdGP131; F and I, AdGP133FLAG. Tunicamycin treated monolayers (G, H and I). For westerns and immunofluorescence assays detection of epitope tagged protein was carried out as described in materials and methods using appropriate mouse primary antibody (anti-myc, anti-HA or anti-FLAG).
(TIF)

S7 Fig RFP-trap immunoprecipitation of GPCMV PC. GPCMV PC IP via RFP trap directed to gLmCherry. **(i)- (ii)** Western blot analysis of immunoprecipitated GPCMV PC. Immunoprecipitation via RFP trap directed to gLmCherry in cells transduced with defective recombinant Ad vectors for all PC components as described in materials and methods. Immunoprecipitated proteins detected by western blot using epitope specific antibodies. Lanes: 1–4, anti-GFP for gH; 5–7, anti mCherry for gL; 8–11, anti-MYC for GP129 12–14, anti-HA for GP131; 15–17, anti-FLAG for GP133. Lanes 2, 5, 9 12 and 16 are total cell lysate. Lanes 4, 7, 11 14 and 17 are immunoprecipitation elusion. Lanes 3, 6, 10 13 and 16 are post bind wash. Lanes 1 and 8 are mock infected total cell lysate samples. **(iii)-(iv)** Control IP of the pentameric complex in the absence of gLmCherry with substituted mCherry control in the presence of gH, GP129, GP131, GP133. Control IP western blot lanes: 1–4, anti-GFP; 5–7, anti-mCherry (for mCherry); 8–11, anti-MYC; 12–14, anti-HA; 15–17, anti-FLAG. Total cell lysate (lanes 2, 5, 9, 12 and 15). Post bind wash flow through (lanes 3, 6, 10, 13, 16). Immunoprecipitation elusion (lanes 4, 7, 11, 14 and 17). Mock infected total cell lysate (lanes 1 and 8).
(TIF)

S8 Fig BLAST alignment of GP129 with GP129C-terminal mutants. A BLAST alignment (MacVector) of the C-terminal regions of the mutant GP129 (NRD13 and GP129UL128) in alignment with wild type GP129 predicted amino acid sequence. NRD13 is a naturally selected GP129 mutation found in the second generation GPCMV BAC GP129UL128 is a C-terminal deletion mutant which encodes 48 codons from the C-terminal of UL128 HCMV (Merlin).
(TIF)

S9 Fig Triplex formation immunoprecipitation assays (full length blots). GP129, GP131, GP133 and gO were evaluated for an ability to form triplex complexes with gH and gL. Transient expression of epithelial cells with gHGFP, gLmCherry, GP129myc, GP131HA and GP133FLAG was as described in materials and methods. Evaluation of triplex formation was by cellular colocalization or by GFP trap immunoprecipitation assay (see Fig 3). This Figure shows the full length western blots of the results from Fig 3. Western blots of triplex immunoprecipitations: (E) gHGFP/gLmCherry/GP129myc; (J) gHGFP/gLmCherry/GP131HA triplex; (O) gHGFP/gLmCherry/GP133FLAG triplex; (T) gHGFP/gLmCherry/gOFLAG triplex. Lanes: 1, 4 and 7 total cell lysate; 2, 5 and 8 wash flow through; 3, 6 and 9 immunoprecipitation. Specific proteins detected indicated in brackets under each blot. Detection by appropriate primary antibody: gHGFP (anti-GFP); gLmCherry (anti-mCherry); GP129myc (anti-myc); GP131 (anti-HA); GP133 (anti-FLAG).
(TIF)

S10 Fig *GP25/GP26* locus sequence of GPCMV and unique *Bam* HI site. GPCMV GP25-26 sequences based on GenBank Accession # AB592928.1. GP25 flanking sequence (yellow); GP26 flanking sequence (green). Unique *Bam*H I site (bold/blue) for SV40 promoter/ SV40 polyA insertion site. Stop codons for GP25 and GP26 are in red text.
(TIF)

S11 Fig Generation of a *GP25/GP26* locus shuttle vector and insertion of a GP129myc cDNA under SV40 promoter control in an ectopic location in GPCMV. **(i)** Location of the GP26/GP26 locus in the GPCMV Hind III genome map. Enlarged area shows the GP25/GP26 region amplified by PCR (genome co-ordinates 37,672–38953) using GP25/GP26 primers (S1 Table) and cloned into pUC19 as a EcoRI/ HindIII fragment of 1.3 kb in size to generate pUCGP25/26. The BamHI site in the intergenic site was further modified. **(ii)** A SV40 promoter/ poly A cassette was inserted to generate pSIGP25/26 (1). Next the GP129myc cDNA was cloned under SV40 control to generate pGP129Link1 (2). A Km cassette was then introduced downstream of the SV40 polyA sequence to generate pGP129LinkKmFRT (3). This shuttle vector was used for recombination with the GPCMV BAC as described in materials and methods. Modified GP25/GP26 locus in the GPCMV BAC genome could be identified by PCR (3.7 kb PCR). The GPCMV BAC locus could be further modified to remove the Km cassette by FLP recombinase (4). **(iii)** PCR analysis of various GPCMV BAC mutants described in S12 Fig with modified GP25/GP26 locus. PCR analysis with *GP25/GP26* primers (S1 Table). PCR products analyzed by agarose gel electrophoresis. Lanes: 1) kb ladder (NEB); 2) NRD13 (wt GP25/GP26 locus); 3) Modified NRD13 locus encoding GP129 and Km (see diagram 3 section (ii) S11 Fig); 4) Modified NRD13 locus encoding GP129 with Km excised (see diagram 4 section (ii). BAC and virus generated GP129FRT; 5) GP128 mutant; 6) GP131 mutant; 7) GP133 mutant; 8) GP129FRT/GP74Km; 9) GP129FRT/GP55Km.
(TIF)

S12 Fig Restriction Profile Analysis GPCMV BAC mutants. Wild type GPCMV BAC was mutated to generate a series of *GP128-GP133* mutants. At least two independent mutants were analyzed per gene knockout but only one mutant is shown in the Figure. Both *Eco*R I (Ec) and *Hind* III (Hd) restriction profile analysis were performed for each mutant but only one profile is shown for each mutant to reduce repetition. Specific band shifts are indicated as original wild type band (yellow) and modified mutant band (red). (i)-(iii) Restriction profiles of individual mutants. Lanes: 1, mutant; 2, wild type GPCMV BAC. Profiles: (i) GP128Km; (ii) GP129-131Km; (iii) GP133Km. (iv)-(v) Restriction profile of double mutant GP128FRT/GP129LinkKm (iv) and GP129-131FRT/GP129LinkKm (v). Double mutants contain an additional modification with an ectopic insertion of a GP129myc cDNA into the *GP25/GP26* intergenic locus. Profiles compared to wild type GPCMV BAC or GP129FRTKm GPCMV BAC (GP129 insertion into the *GP25/GP26* locus). BAC profiles. Lanes: 1, double mutant; 2, GP129FRTKm; 3, wild type GPCMV. Modified *GP25/GP26* locus indicated. (vi) Generation of GP74 knockout mutant on a GP129 positive background. Restriction profile of NBGP129FRT/GP74Km (lane 1) vs wild type GPCMV BAC (lane 2). Modified GP74 locus indicated. (vii) Generation of GP55 mutant on a GP129 positive background. GPCMV BAC EcoRI profile analysis: Lanes: 1, GP129FRT/GP55km; 2, GP129FRT; 3, NRD13 (original BAC). Wild type band indicated (yellow dot) and modified band indicated (red dot).
(TIF)

S13 Fig Growth curves of *GP128-GP133* locus mutants on GPL cells and tropism of GP131 and GP133 mutants for epithelial cells. **(i)** Growth curve of various GPCMV *GP128-GP133* mutants on GPL cells. Moi per mutant virus was 1pfu/cell. Samples taken at 1–10 days post infection and titrated in duplicate on GPL cells as described in materials and methods. GPCMV *GP128-133* locus mutants: *GP128* mutant (GP128FRT/GP129Link); *GP133* mutant (GP133FRT/GP129Link); *GP131* mutant (GP131FRT/GP129Link); *GP129* mutant (NRD13). **(ii)** Comparative growth of *GP131* and *GP133* mutants on GPL and EPI cells. GPL (panels A and C) and EPI (panels B and D) cells were infected at a moi of 1pfu/cell with respective mutant viruses: GP133 mutant, panels A & B; GP131 mutant, panels C & D. Virus growth on cells evaluated at approximately 3 days post infection for GFP reporter gene expression and cells counter stained for cell nuclei with DAPI. Growth curve study carried out with Cre treated BAC excised virus stocks and virus GFP fluorescence studies (ii) carried out with GFP+ non cre excised virus stocks.
(TIF)

S14 Fig Both PC and gB are required for GPCMV infection of epithelial cells. PC+/gO+/ gB negative GPCMV BAC mutant (GP129FRT/GP55Km) transfected onto epithelial cells failed to produce infectious virus (**A**). Single transfected cells identified by GFP reporter gene expression; gB rescue (GP129FRT/GP55Km rescue). Mutant gB BAC (GP129FRT/GP55km) was co-transfected onto GPL cells with a GP55 rescue fragment to restore epithelial tropism. Virus spread detected by GFP reporter gene expression at day 12 post transfection restored (**B**).
(TIF)

S15 Fig Inhibition of GPCMV epithelial infection via Bafilomycin A1 treatment. EPI or GPL cells were untreated or pretreated with 50nM bafilomycin A1 for 1 hr prior to GPCMVGP129FRT virus infection (MOI = 1pfu/cell). **(i)** Virus infection of EPI cells in presence or absence of bafilomycin. Images of random cell fields immunostained for cytokeratin and GPCMV IE2 protein. Images of cells: mock infected EPI cells treated with 50nM bafilomycin A1 (images A-C); untreated EPI cells infected with FRT virus (images D-F); pretreated with 50nM bafilomycin A1(images G-I). Cells immunostained as described in materials and methods: anti-pan-keratin antibody with secondary anti-mouse IgG-TritC (A, D, and G) and anti-IE2 antibody with secondary anti-rabbit IgG-FitC (B, E, and H). Cells were counter stained with DAPI (C, F and I). Images were taken at 40X magnification using spinning disc confocal microscope (Olympus). **(ii)** Percentage of GPCMV infected cells (GPL or EPI) either bafilomycin treated or untreated. Virus detected by IE2 antigen expression via immunofluorescence assay using anti-IE2 antibody as described in materials and methods. Thirty random fields for each 3 independent experiments per treatment were counted. Statistical analysis was performed with student t-test.
(TIF)
