# Supplementary material for: A Homolog Pentameric Complex Dictates Viral Epithelial Tropism, Pathogenicity and Congenital Infection Rate in Guinea Pig Cytomegalovirus
Source: PLoS Pathog. 2016 Jul 7;12(7):e1005755. doi: 10.1371/journal.ppat.1005755 (PMC4936736; doi:10.1371/journal.ppat.1005755)
Supplement: S1 Table — (DOCX) [file ppat.1005755.s001.docx]

**S1 Table. Oliogonucleotides used for PCR and RT-PCR analysis.**

| **Primer name** | **Sequence** |
| --- | --- |
| P1 | 5’GTAGGTACCCGCAGGTTTGC |
| P2 | 5’TTGATCACGGACGACGATAC |
| FGP25 | 5’GACAATCTGTGCGAACAGGCATACGG |
| RGP26 | 5’GATGCGTGATAGGCATAGCGGAG |
| Link1F | 5’GGAATTCAGATCTGTTTAAACG |
| Link1R | 5’TCGACGTTTAAACAGATCTGAATTCCTGCA |
| GP128F | GGATCCATGAGTCCCGTCGTCAATCGACTGCCCG |
| GP128R | GGATCCGTCGATGGTAACGGGAAGTAACTCCTG |
| FgHBm | 5’GGATCCATGTCACCCGCGACGCGGTTTACC |
| RgHBmNostop | 5’GGATCCAGAATCATATGTAAGAGGCGGTATAGACCGTATAG |
| FgLHd | 5’AAGCTTATGTATGAATGTATGTTTTTTTCGCATCGT |
| RgLHdNostop | 5’AAGCTTGCTCAGATCCCTGTTTAAGTAATTGTG |
| RTGP128F | 5’TCTGGAGACGTCCGCGTTTCG |
| RTGP128R | 5’CAGTCCGATCTGCTCAGAC |
| RTGP129F | 5’GTGTACGCCATGGCGCATAACTG |
| RTGP129R | 5’GATCTACGTTGTTTCATATCCACG |
| RTGP130F | 5’CACTTGTTTGTTAATGCCTACC |
| RTGP130R | 5’CACTCCACCACGTCAAACGATAC |
| RTGP131F | 5’GAGTGACCGATAATACAAG |
| RTGP131R | 5’GTATTGAGCATGTTAACGTAGAG |
| RTGP133F | 5’CGTCAATGCTCACCAGCCCTT |
| RTGP133R | 5’TTTCGATCGGCGCGGAGGATG |
| GAPDHRTF | 5’GGGCAAGGTCATCCCAGAG |
| GAPDHRTR | 5’TGGAAGAATGGCTGTCACTGTT |
