# Supplementary material for: A Homolog Pentameric Complex Dictates Viral Epithelial Tropism, Pathogenicity and Congenital Infection Rate in Guinea Pig Cytomegalovirus
Source: PLoS Pathog. 2016 Jul 7;12(7):e1005755. doi: 10.1371/journal.ppat.1005755 (PMC4936736; doi:10.1371/journal.ppat.1005755)
Supplement: S2 Table — (DOCX) [file ppat.1005755.s002.docx]

**S2 Table. Predicted molecular weights of pentameric complex proteins.**

| **Protein** | **Predicted Size (kDa)** | **Observed Size (kDa)** | **N-glycosylation sites** | **O-glycosylation sites** |
| --- | --- | --- | --- | --- |
| **gH** | 81.8 | 80 | 10 | 10 |
| **gH-GFP** | 108.6 | 115 |  |  |
| **gL** | 29.7 |  | 3 | 3 |
| **gL-mCherry** | 56.4 | 60 |  |  |
| **GP129** | 20.6 |  | 4 | 2 |
| **GP129-MYC** | 24.2 | 40 |  |  |
| **GP131** | 21.8 |  | 2 | 6 |
| **GP131-HA** | 25.1 | 31 |  |  |
| **GP133** | 14.7 |  | 0 | 1 |
| **GP133-FLAG** | 16.7 | 19 |  |  |
| **GP129NRD13-MYC** | 15.4 | 27 | 2 | 2 |
| **GP129UL128-MYC** | 23.8 | 32 | 2 | 5 |

Post translational glycosylation predicted based on web programs: NetOGlyc 4.0 Server (http://www.cbs.dtu.dk/services/NetOGlyc/) for O-glycosylation; and NetNGlyc 1.0 Server (http://www.cbs.dtu.dk/services/NetNGlyc/) for N- glycosylation. Total predicted number of N-glycosylation or O-glycosylation sites per glycoprotein are indicated.
