# Supplementary figures and images for: A Homolog Pentameric Complex Dictates Viral Epithelial Tropism, Pathogenicity and Congenital Infection Rate in Guinea Pig Cytomegalovirus
Source: PLoS Pathog. 2016 Jul 7;12(7):e1005755. doi: 10.1371/journal.ppat.1005755 (PMC4936736; doi:10.1371/journal.ppat.1005755)

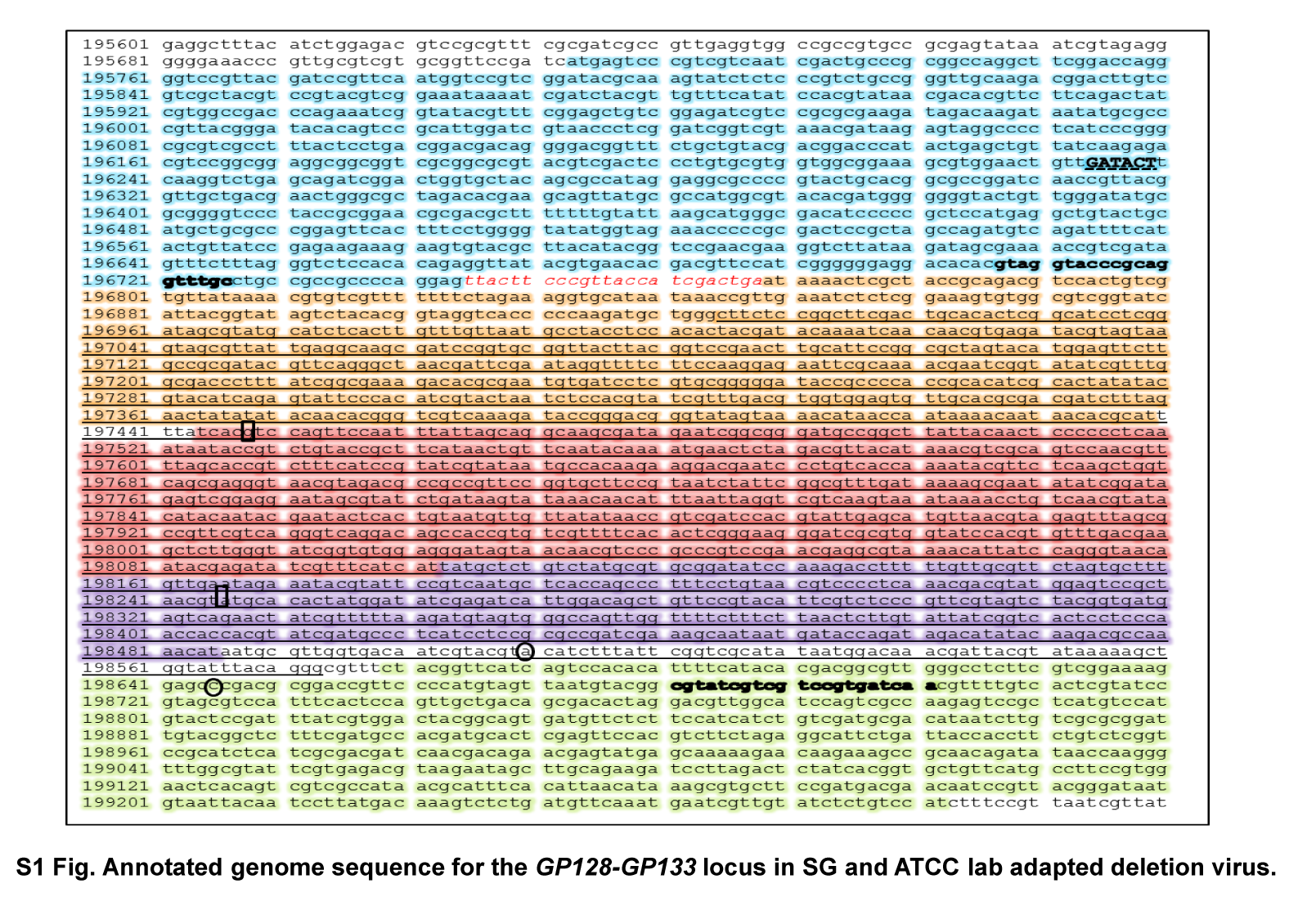

Supplement: S1 Fig — GPCMV GP128-134 sequences based on GenBank Accession # AB592928.1. GPCMV nucleotide base co-ordinates indicated in brackets. GP128 (195,713–196,768) highlighted in blue; GP129 (196,745–197,439 complement) highlighted in green; GP131 (197,444–198,102 complement) highlighted in orange; GP133 (198,102–198,485 complement) highlighted in yellow GP134 (198,579–199,262 complement) highlighted in grey. Primers P1/P2 (S1 Table) used for verification of intact or deleted GP129-133 region are bolded in black. Deleted region (1.6 kb) of lab-adapted virus is underlined (196,925–198,573). Sequence deletion for GP129-GP131 mutant (197,292–198,090) start and end sequence of deletion designated by square (□). Sequence deletion for GP133 mutant (198,361–198,489) start and end sequence designated by circle (○). For the GP128 mutant, an EcoR V site (196,234–196,239) within GP128 was the site of a Km cassette insertion (GATATC) which disrupted the ORF. (TIF) [file ppat.1005755.s003.tif]

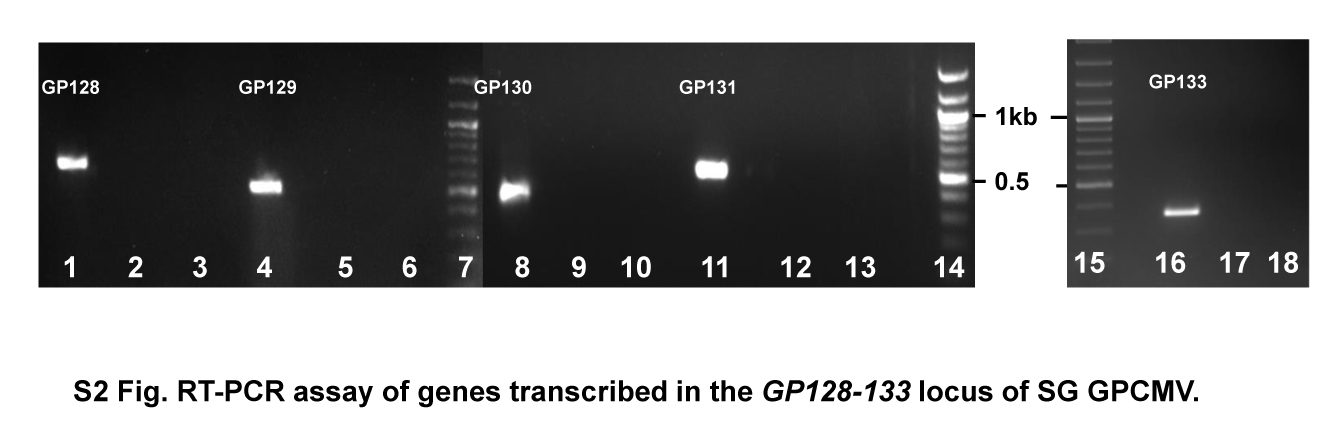

Supplement: S2 Fig — Individual RT-PCR primer sets (see material and methods and S1 Table) were designed for GP128, GP129, GP130, GP131and GP133 based on defined sequences. RT-PCR was performed as previously described [36]. RT-PCR products were analysed by agarose gel electrophoresis. Lanes: 1–3, GP128; 4–6 GP129; 8–10 GP130; 11–13, GP131. Lanes 1, 4, 8, 11 and 16 SG GPCMV infected cells. Lanes 2, 5, 9, 12 and 17 mock infected cells (control). Lanes 3, 5, 9, 12 and 18 no RNA control. Lanes 7,14 and 15,100 bp ladder (NEB). (TIF) [file ppat.1005755.s004.tif]

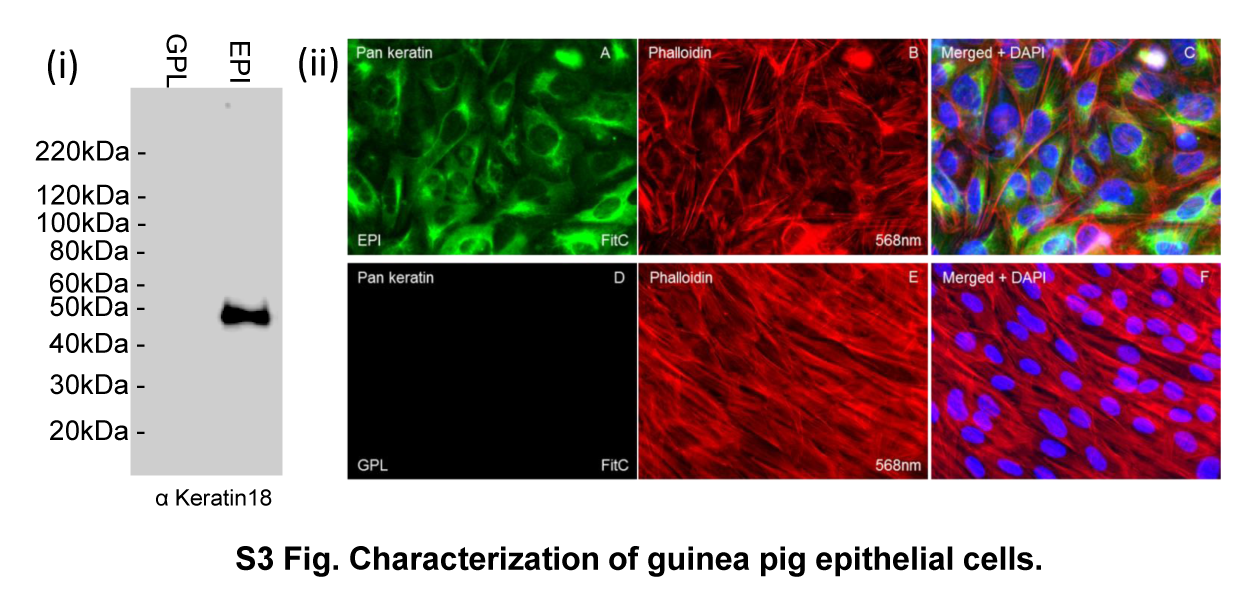

Supplement: S3 Fig — (i). GPL fibroblast vs Epithelial cell western blot for cytokeratin 18. Cell lysates from ~1x10^6^ GPL and EPI cells were analyzed by western blot using a 4–20% SDS-PAGE gel and probed with anti-Keratin 18 (DC10) Mouse mAb (Cell Signaling) and secondary anti-mouse IgG-HRP conjugate. (ii). Immunofluorescence for cytokeratin expression in EPI cells. Monolayers of EPI (images A, B & C) and GPL (images D, E & F) cells were immunostained using Pan-Keratin (C11) mouse mAb (Images A and D) as described in materials and methods. Cell were also stained with high-affinity F-actin probe, anti-phalloidin-Alexa Fluor 568 (ThermoFisher scientific) (Images B and E). Cells were counterstained with DAPI (merged images C and F). Images were taken at 40X using a Olympus IX81 confocal microscope. (TIF) [file ppat.1005755.s005.tif]

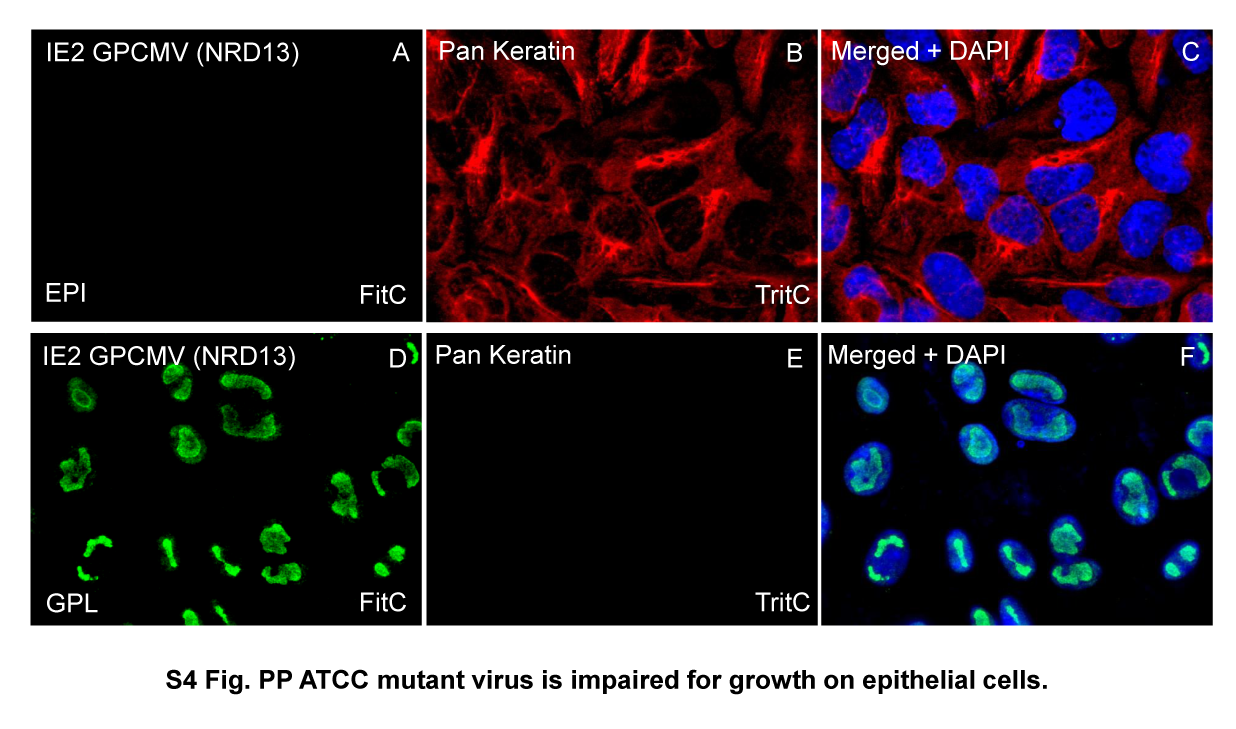

Supplement: S4 Fig — EPI and GPL cells were infected at a moi of 1 pfu/cell. At 48 hr post infection cells were fixed and stained for viral (IE2) and epithelial cell markers as described in materials and methods. Immunofluorescence images of EPI cells: A, IE2; B, pan-keratin; C, merged A and B with DAPI stain. Immunofluorescence images of GPL cells: D, IE2; E, pan-keratin; F, merged E and F with DAPI stain. Images at x60 maginification. (TIF) [file ppat.1005755.s006.tif]

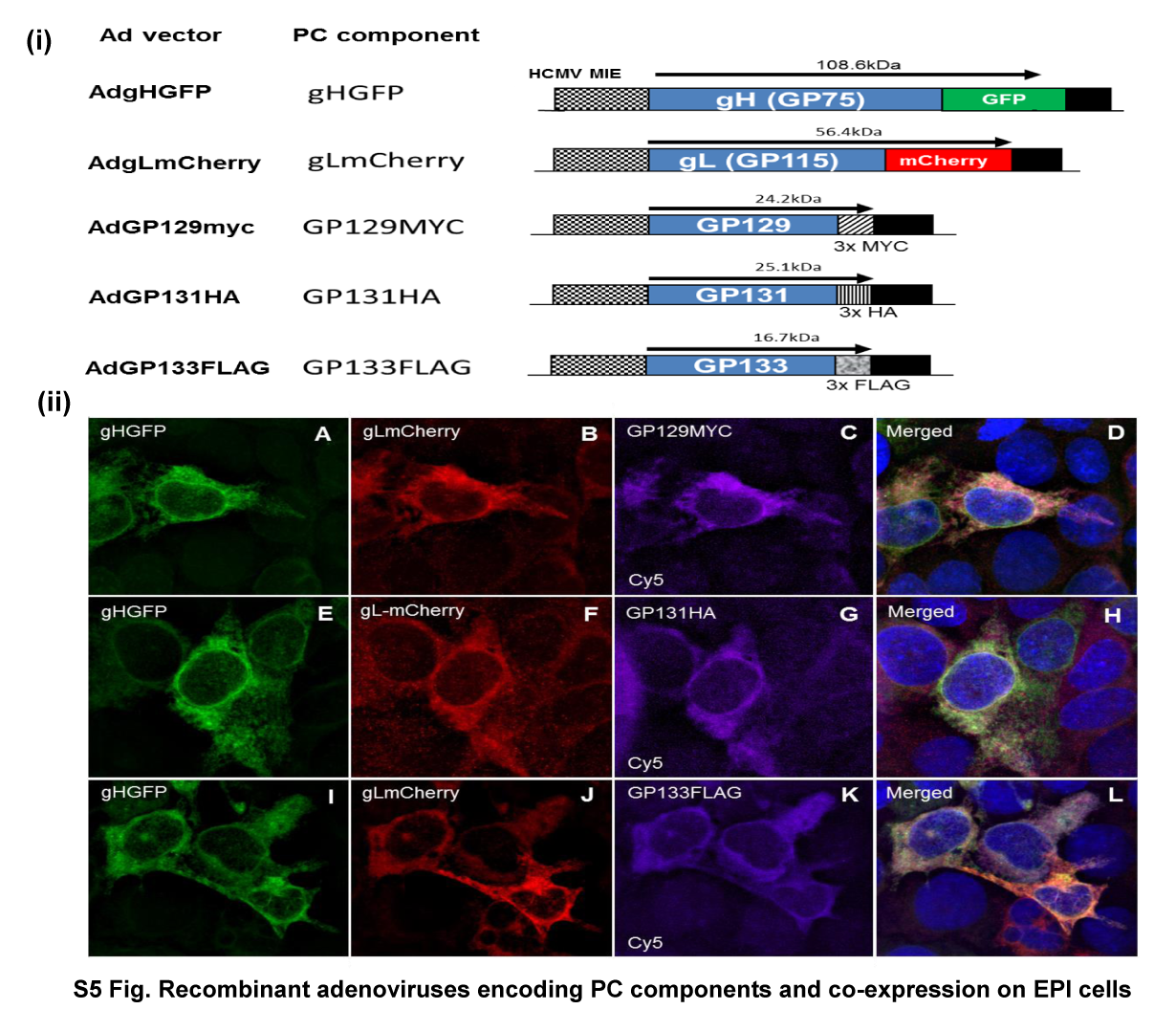

Supplement: S5 Fig — (i) Components of the pentameric complex (gH, gL. GP129, GP131 and GP133) were individually cloned as C-terminal epitope tagged ORFs into recombinant defective adenovirus shuttle vectors and recombinant viruses generated for each component. Genes were expressed under HCMV MIE enhancer expression as illustrated. Predicted encoded protein is indicated for each construct. (ii) Cellular co-localization of pentameric complex components in guinea pig epithelial cells in the absence of other GPCMV proteins. EPI cells were transduced with defective recombinant Ad constructs of the pentameric complex as described in materials and methods. gH expression detected under fluorescence (gHGFP). gHGFP localization (panels A, E, and I), gL expression detected under fluorescence (gLmCherry). gLmCherry localization (panels B, F, & J). GP129myc localization using immunofluorescence anti–myc antibody/ Cy5 (Panel C). GP131HA localization using anti-HA antibody/Cy5 (G). GP133FLAG localization using anti-FLAG antibody/Cy5 (K). Panel D (merged A, B & C) for gH, gL and GP129, Panel H (merged E, F & G) for gH, gL and GP131. Panel L (merged I, J & K) for gH, gL and GP133. Merged images also counterstained with DAPI. (TIF) [file ppat.1005755.s007.tif]

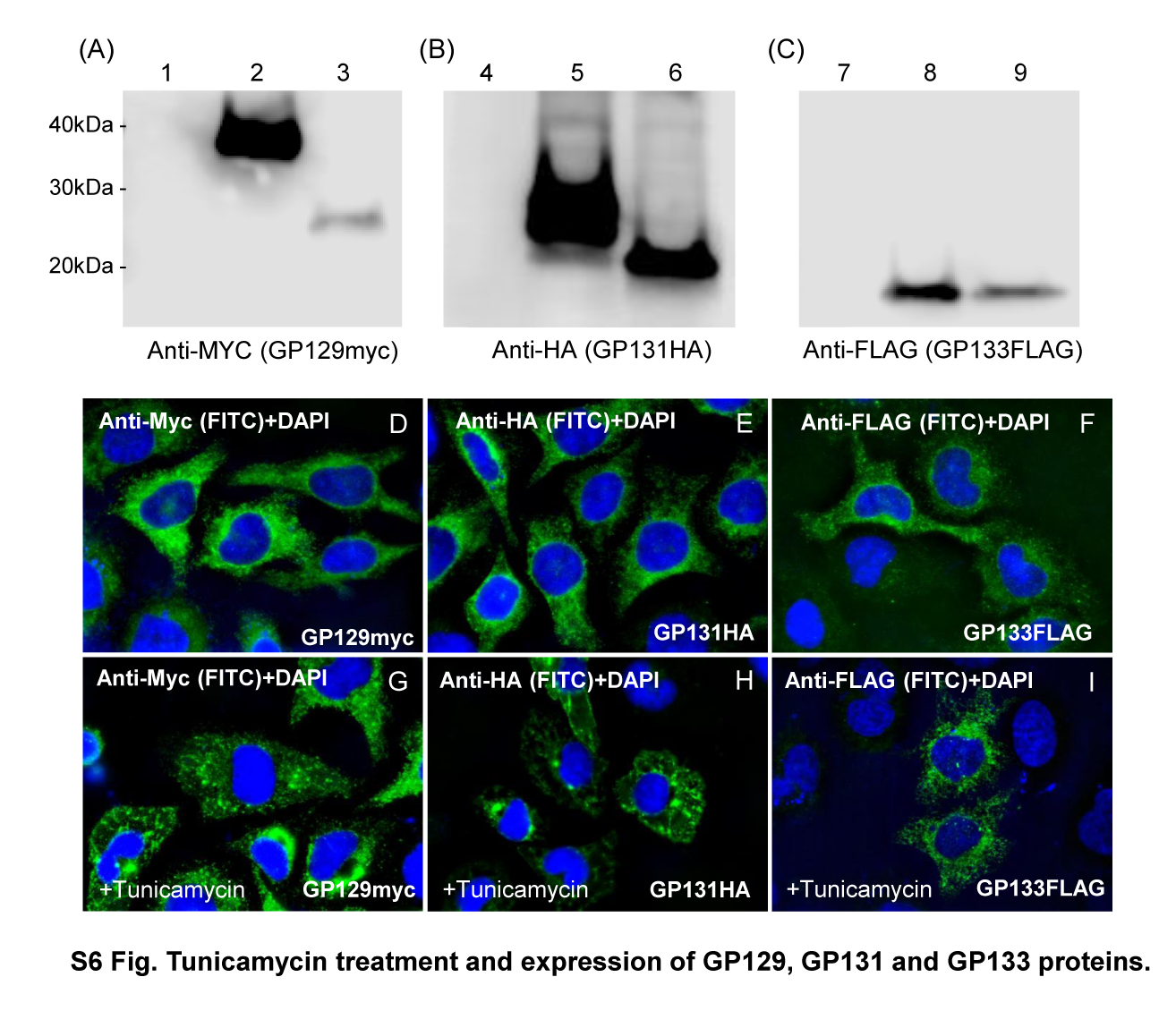

Supplement: S6 Fig — Transient expression of GP129, GP131 and GP133 was evaluated in the presence or absence of tunicamycin treatment. Separate 6 well plates of epithelial cells were transduced with recombinant Ad vectors encoding GP129, GP131 or GP133. Expression occurred in the presence or absence of tunicamycin as previously described (36). After overnight expression, monolayers were either harvested for western blot analysis (A-C) or fixed for immunofluorescence assay (D-I). Western blot assays (A-C). Lanes: 1, 4 and 7 mock infected cells; 2 and 3 AdGP129myc transduced cells; 5 and 6 AdGP131HA; 8 and 9 AdGP133FLAG. Lanes 3, 6 and 9 represent cells treated with tunicamycin. Immunofluorescence assays: D and G, AdGP129myc; E and H, AdGP131; F and I, AdGP133FLAG. Tunicamycin treated monolayers (G, H and I). For westerns and immunofluorescence assays detection of epitope tagged protein was carried out as described in materials and methods using appropriate mouse primary antibody (anti-myc, anti-HA or anti-FLAG). (TIF) [file ppat.1005755.s008.tif]

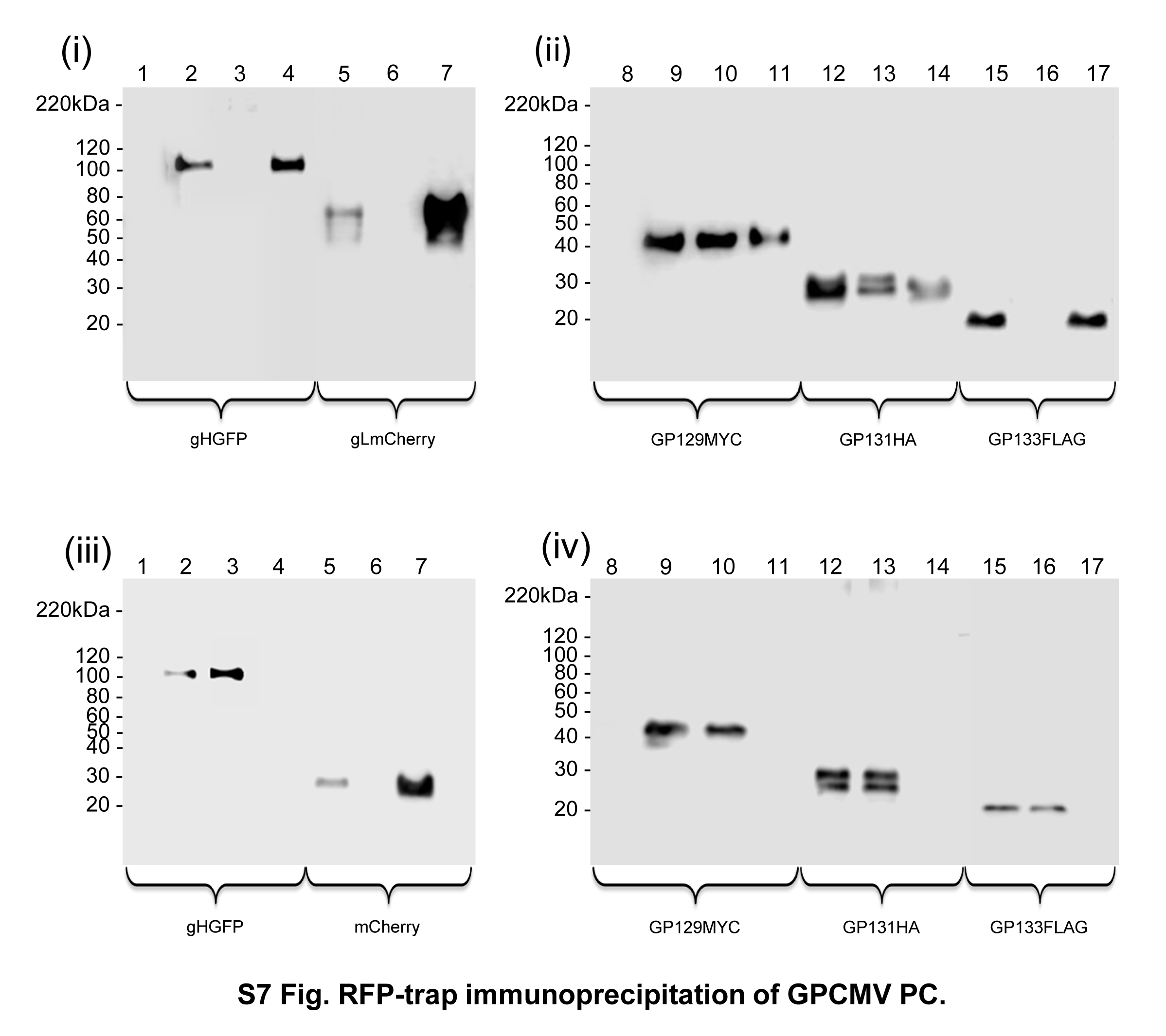

Supplement: S7 Fig — GPCMV PC IP via RFP trap directed to gLmCherry. (i)- (ii) Western blot analysis of immunoprecipitated GPCMV PC. Immunoprecipitation via RFP trap directed to gLmCherry in cells transduced with defective recombinant Ad vectors for all PC components as described in materials and methods. Immunoprecipitated proteins detected by western blot using epitope specific antibodies. Lanes: 1–4, anti-GFP for gH; 5–7, anti mCherry for gL; 8–11, anti-MYC for GP129 12–14, anti-HA for GP131; 15–17, anti-FLAG for GP133. Lanes 2, 5, 9 12 and 16 are total cell lysate. Lanes 4, 7, 11 14 and 17 are immunoprecipitation elusion. Lanes 3, 6, 10 13 and 16 are post bind wash. Lanes 1 and 8 are mock infected total cell lysate samples. (iii)-(iv) Control IP of the pentameric complex in the absence of gLmCherry with substituted mCherry control in the presence of gH, GP129, GP131, GP133. Control IP western blot lanes: 1–4, anti-GFP; 5–7, anti-mCherry (for mCherry); 8–11, anti-MYC; 12–14, anti-HA; 15–17, anti-FLAG. Total cell lysate (lanes 2, 5, 9, 12 and 15). Post bind wash flow through (lanes 3, 6, 10, 13, 16). Immunoprecipitation elusion (lanes 4, 7, 11, 14 and 17). Mock infected total cell lysate (lanes 1 and 8). (TIF) [file ppat.1005755.s009.tif]

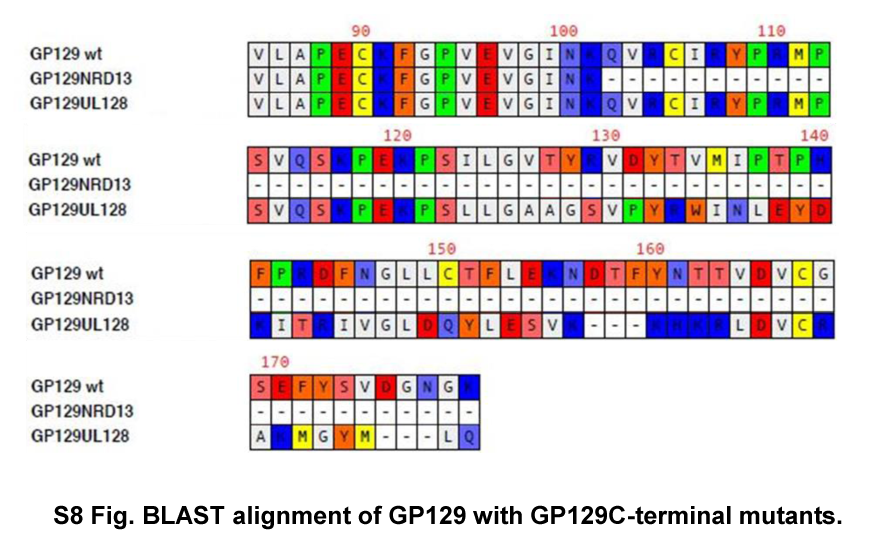

Supplement: S8 Fig — A BLAST alignment (MacVector) of the C-terminal regions of the mutant GP129 (NRD13 and GP129UL128) in alignment with wild type GP129 predicted amino acid sequence. NRD13 is a naturally selected GP129 mutation found in the second generation GPCMV BAC GP129UL128 is a C-terminal deletion mutant which encodes 48 codons from the C-terminal of UL128 HCMV (Merlin). (TIF) [file ppat.1005755.s010.tif]

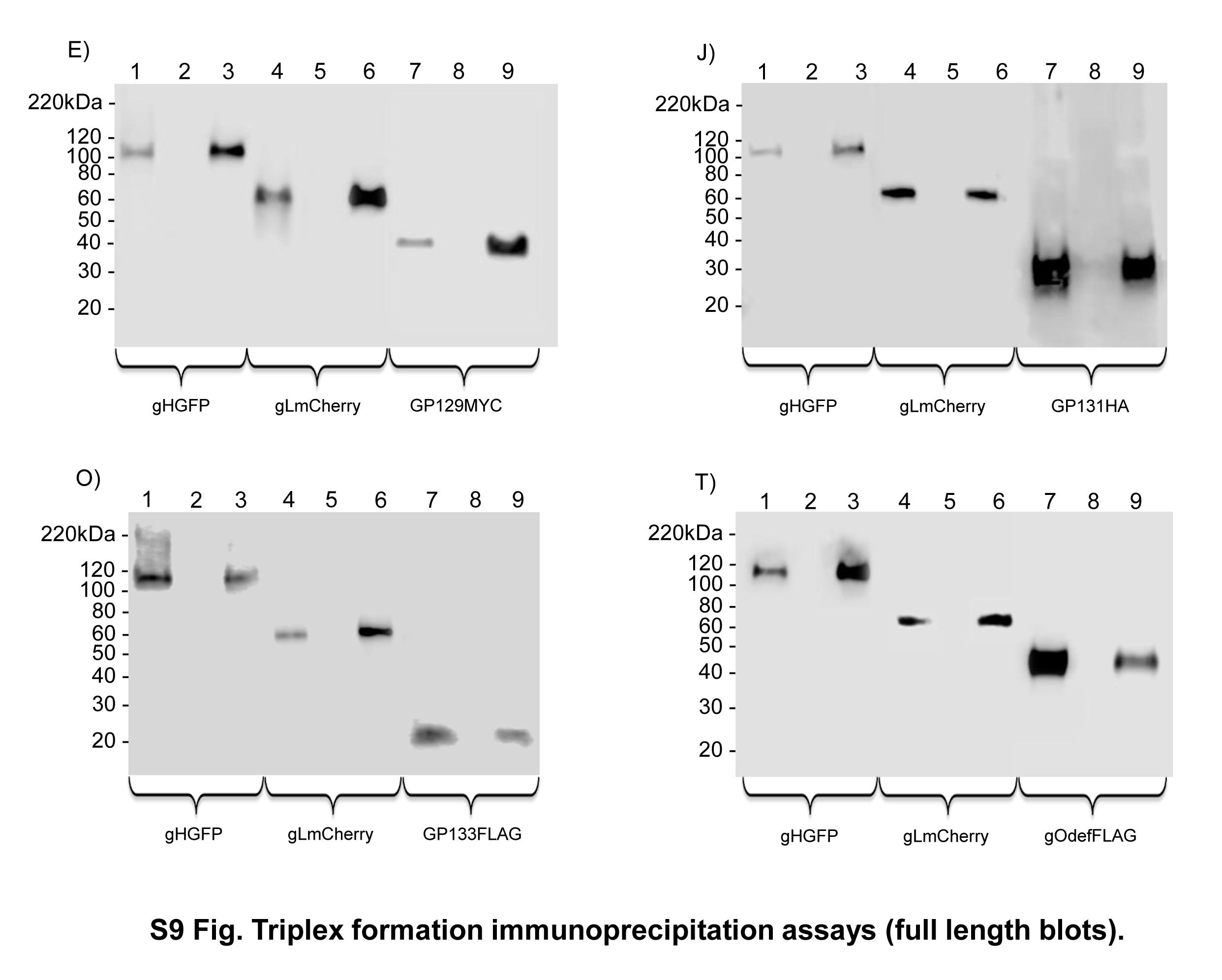

Supplement: S9 Fig — GP129, GP131, GP133 and gO were evaluated for an ability to form triplex complexes with gH and gL. Transient expression of epithelial cells with gHGFP, gLmCherry, GP129myc, GP131HA and GP133FLAG was as described in materials and methods. Evaluation of triplex formation was by cellular colocalization or by GFP trap immunoprecipitation assay (see Fig 3). This Figure shows the full length western blots of the results from Fig 3. Western blots of triplex immunoprecipitations: (E) gHGFP/gLmCherry/GP129myc; (J) gHGFP/gLmCherry/GP131HA triplex; (O) gHGFP/gLmCherry/GP133FLAG triplex; (T) gHGFP/gLmCherry/gOFLAG triplex. Lanes: 1, 4 and 7 total cell lysate; 2, 5 and 8 wash flow through; 3, 6 and 9 immunoprecipitation. Specific proteins detected indicated in brackets under each blot. Detection by appropriate primary antibody: gHGFP (anti-GFP); gLmCherry (anti-mCherry); GP129myc (anti-myc); GP131 (anti-HA); GP133 (anti-FLAG). (TIF) [file ppat.1005755.s011.tif]

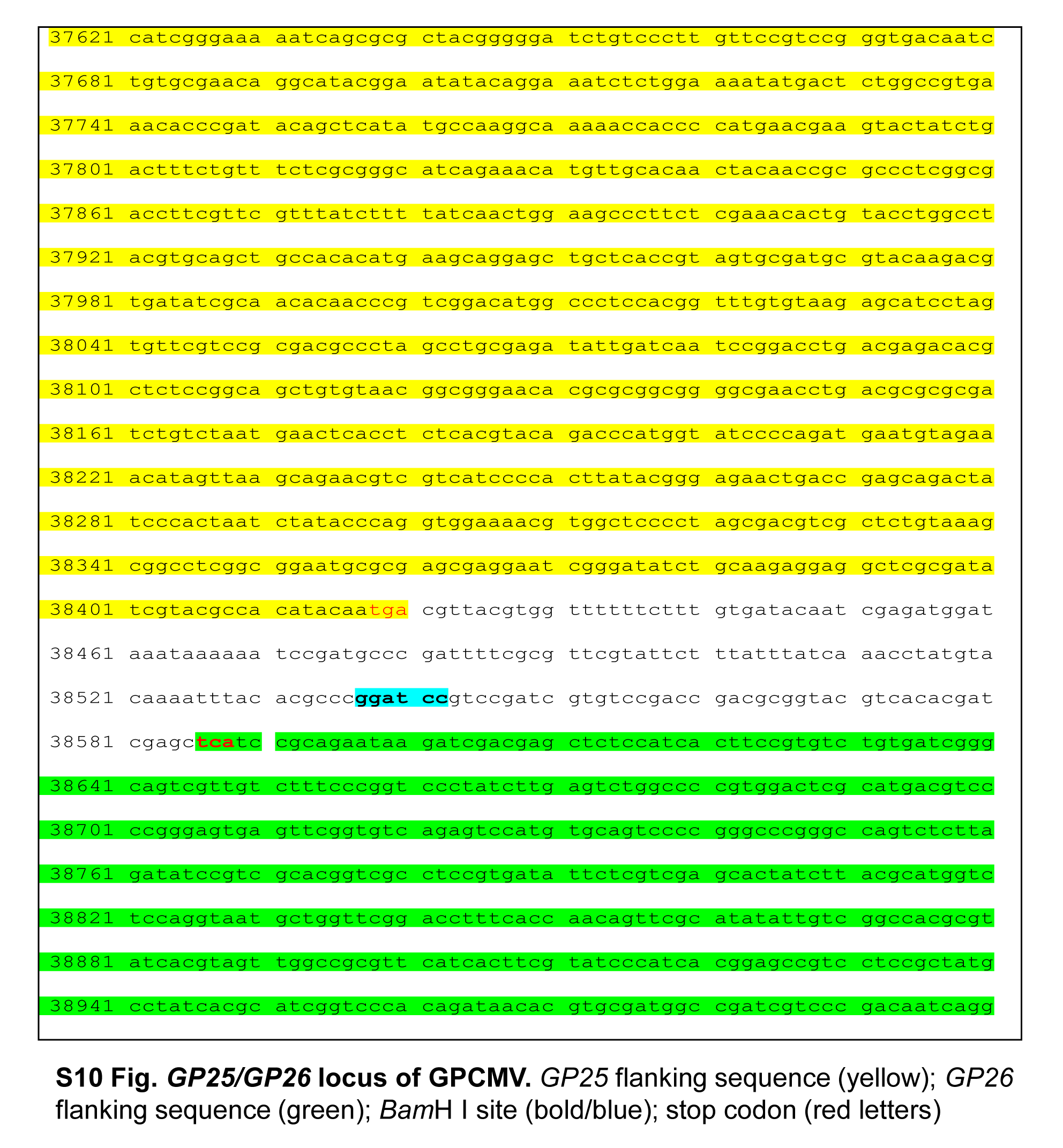

Supplement: S10 Fig — GPCMV GP25-26 sequences based on GenBank Accession # AB592928.1. GP25 flanking sequence (yellow); GP26 flanking sequence (green). Unique BamH I site (bold/blue) for SV40 promoter/ SV40 polyA insertion site. Stop codons for GP25 and GP26 are in red text. (TIF) [file ppat.1005755.s012.tif]

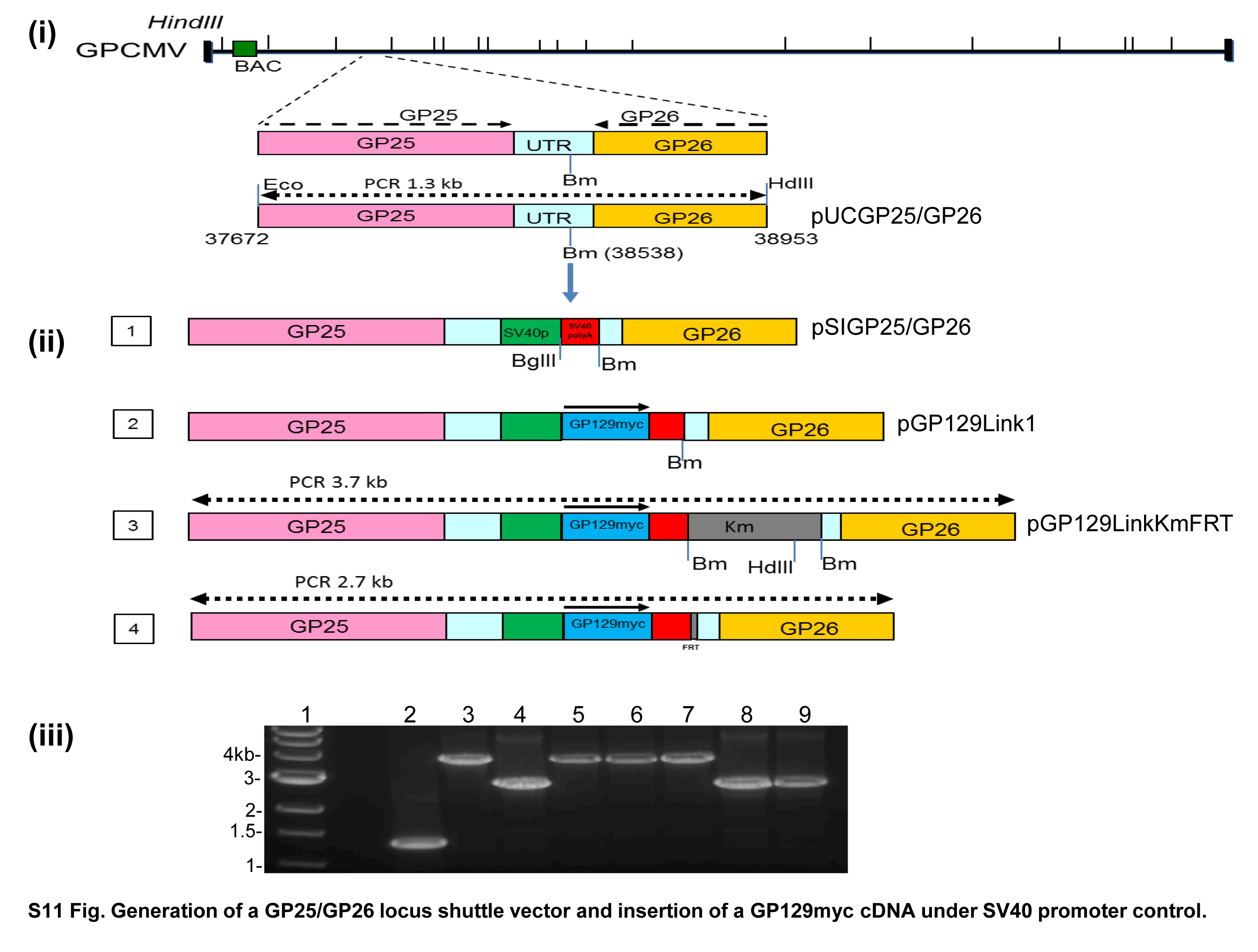

Supplement: S11 Fig — (i) Location of the GP26/GP26 locus in the GPCMV Hind III genome map. Enlarged area shows the GP25/GP26 region amplified by PCR (genome co-ordinates 37,672–38953) using GP25/GP26 primers (S1 Table) and cloned into pUC19 as a EcoRI/ HindIII fragment of 1.3 kb in size to generate pUCGP25/26. The BamHI site in the intergenic site was further modified. (ii) A SV40 promoter/ poly A cassette was inserted to generate pSIGP25/26 (1). Next the GP129myc cDNA was cloned under SV40 control to generate pGP129Link1 (2). A Km cassette was then introduced downstream of the SV40 polyA sequence to generate pGP129LinkKmFRT (3). This shuttle vector was used for recombination with the GPCMV BAC as described in materials and methods. Modified GP25/GP26 locus in the GPCMV BAC genome could be identified by PCR (3.7 kb PCR). The GPCMV BAC locus could be further modified to remove the Km cassette by FLP recombinase (4). (iii) PCR analysis of various GPCMV BAC mutants described in S12 Fig with modified GP25/GP26 locus. PCR analysis with GP25/GP26 primers (S1 Table). PCR products analyzed by agarose gel electrophoresis. Lanes: 1) kb ladder (NEB); 2) NRD13 (wt GP25/GP26 locus); 3) Modified NRD13 locus encoding GP129 and Km (see diagram 3 section (ii) S11 Fig); 4) Modified NRD13 locus encoding GP129 with Km excised (see diagram 4 section (ii). BAC and virus generated GP129FRT; 5) GP128 mutant; 6) GP131 mutant; 7) GP133 mutant; 8) GP129FRT/GP74Km; 9) GP129FRT/GP55Km. (TIF) [file ppat.1005755.s013.tif]

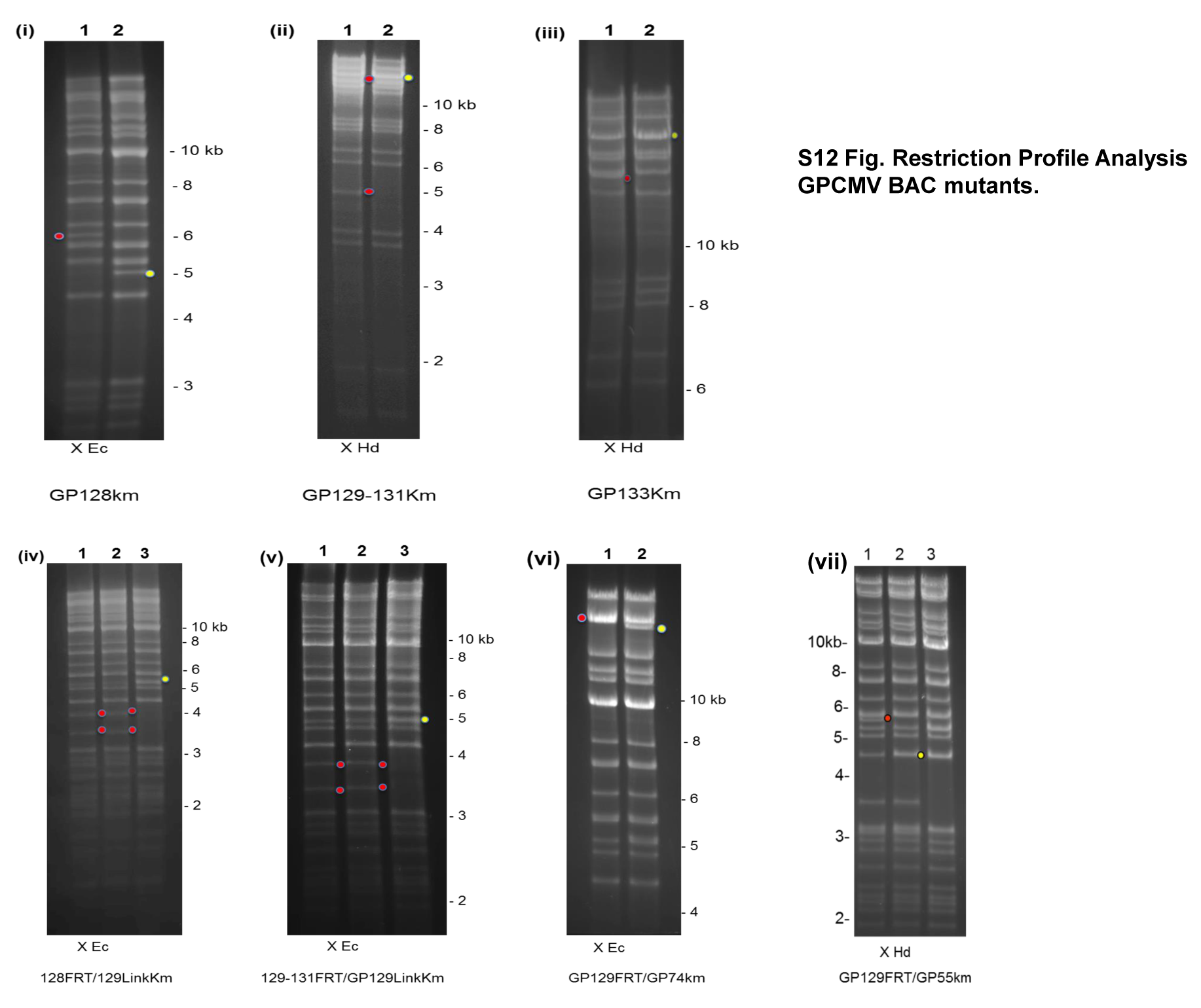

Supplement: S12 Fig — Wild type GPCMV BAC was mutated to generate a series of GP128-GP133 mutants. At least two independent mutants were analyzed per gene knockout but only one mutant is shown in the Figure. Both EcoR I (Ec) and Hind III (Hd) restriction profile analysis were performed for each mutant but only one profile is shown for each mutant to reduce repetition. Specific band shifts are indicated as original wild type band (yellow) and modified mutant band (red). (i)-(iii) Restriction profiles of individual mutants. Lanes: 1, mutant; 2, wild type GPCMV BAC. Profiles: (i) GP128Km; (ii) GP129-131Km; (iii) GP133Km. (iv)-(v) Restriction profile of double mutant GP128FRT/GP129LinkKm (iv) and GP129-131FRT/GP129LinkKm (v). Double mutants contain an additional modification with an ectopic insertion of a GP129myc cDNA into the GP25/GP26 intergenic locus. Profiles compared to wild type GPCMV BAC or GP129FRTKm GPCMV BAC (GP129 insertion into the GP25/GP26 locus). BAC profiles. Lanes: 1, double mutant; 2, GP129FRTKm; 3, wild type GPCMV. Modified GP25/GP26 locus indicated. (vi) Generation of GP74 knockout mutant on a GP129 positive background. Restriction profile of NBGP129FRT/GP74Km (lane 1) vs wild type GPCMV BAC (lane 2). Modified GP74 locus indicated. (vii) Generation of GP55 mutant on a GP129 positive background. GPCMV BAC EcoRI profile analysis: Lanes: 1, GP129FRT/GP55km; 2, GP129FRT; 3, NRD13 (original BAC). Wild type band indicated (yellow dot) and modified band indicated (red dot). (TIF) [file ppat.1005755.s014.tif]

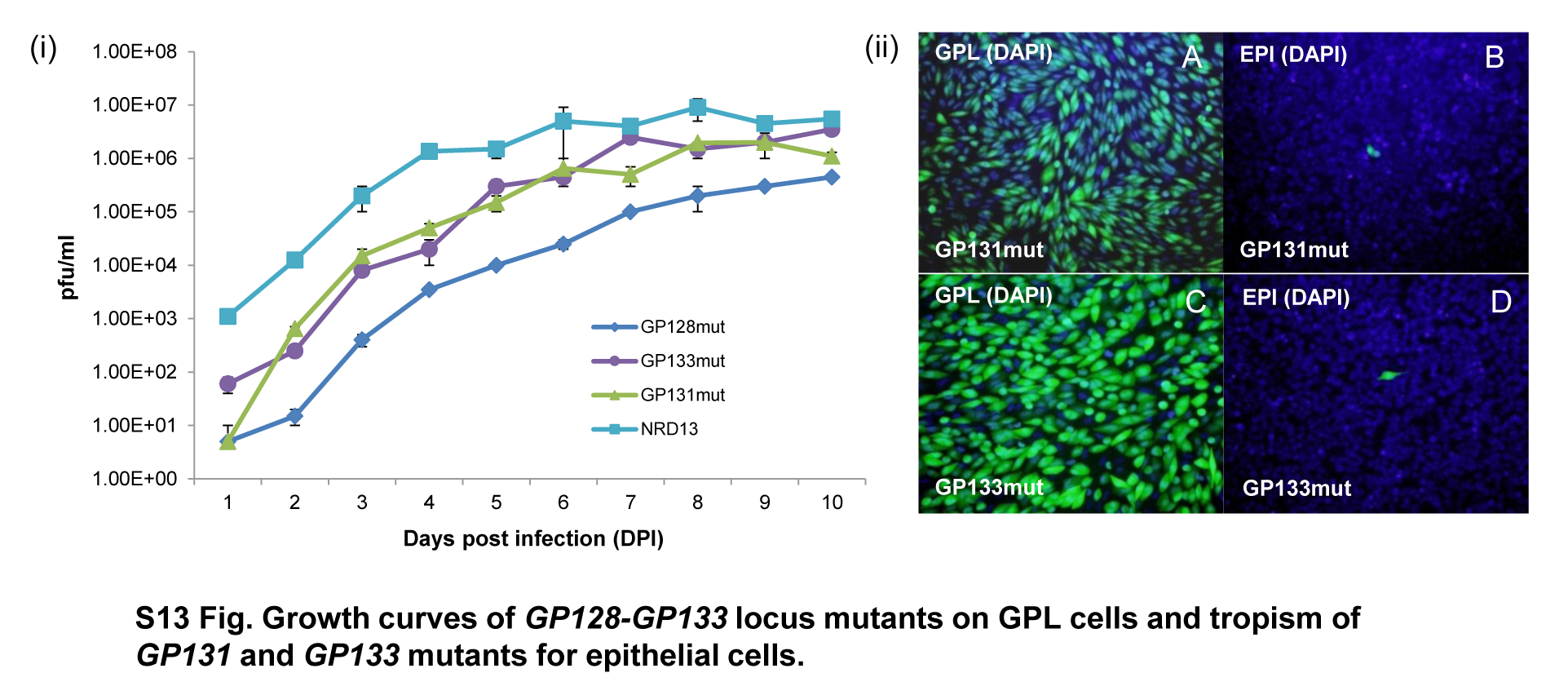

Supplement: S13 Fig — (i) Growth curve of various GPCMV GP128-GP133 mutants on GPL cells. Moi per mutant virus was 1pfu/cell. Samples taken at 1–10 days post infection and titrated in duplicate on GPL cells as described in materials and methods. GPCMV GP128-133 locus mutants: GP128 mutant (GP128FRT/GP129Link); GP133 mutant (GP133FRT/GP129Link); GP131 mutant (GP131FRT/GP129Link); GP129 mutant (NRD13). (ii) Comparative growth of GP131 and GP133 mutants on GPL and EPI cells. GPL (panels A and C) and EPI (panels B and D) cells were infected at a moi of 1pfu/cell with respective mutant viruses: GP133 mutant, panels A & B; GP131 mutant, panels C & D. Virus growth on cells evaluated at approximately 3 days post infection for GFP reporter gene expression and cells counter stained for cell nuclei with DAPI. Growth curve study carried out with Cre treated BAC excised virus stocks and virus GFP fluorescence studies (ii) carried out with GFP+ non cre excised virus stocks. (TIF) [file ppat.1005755.s015.tif]

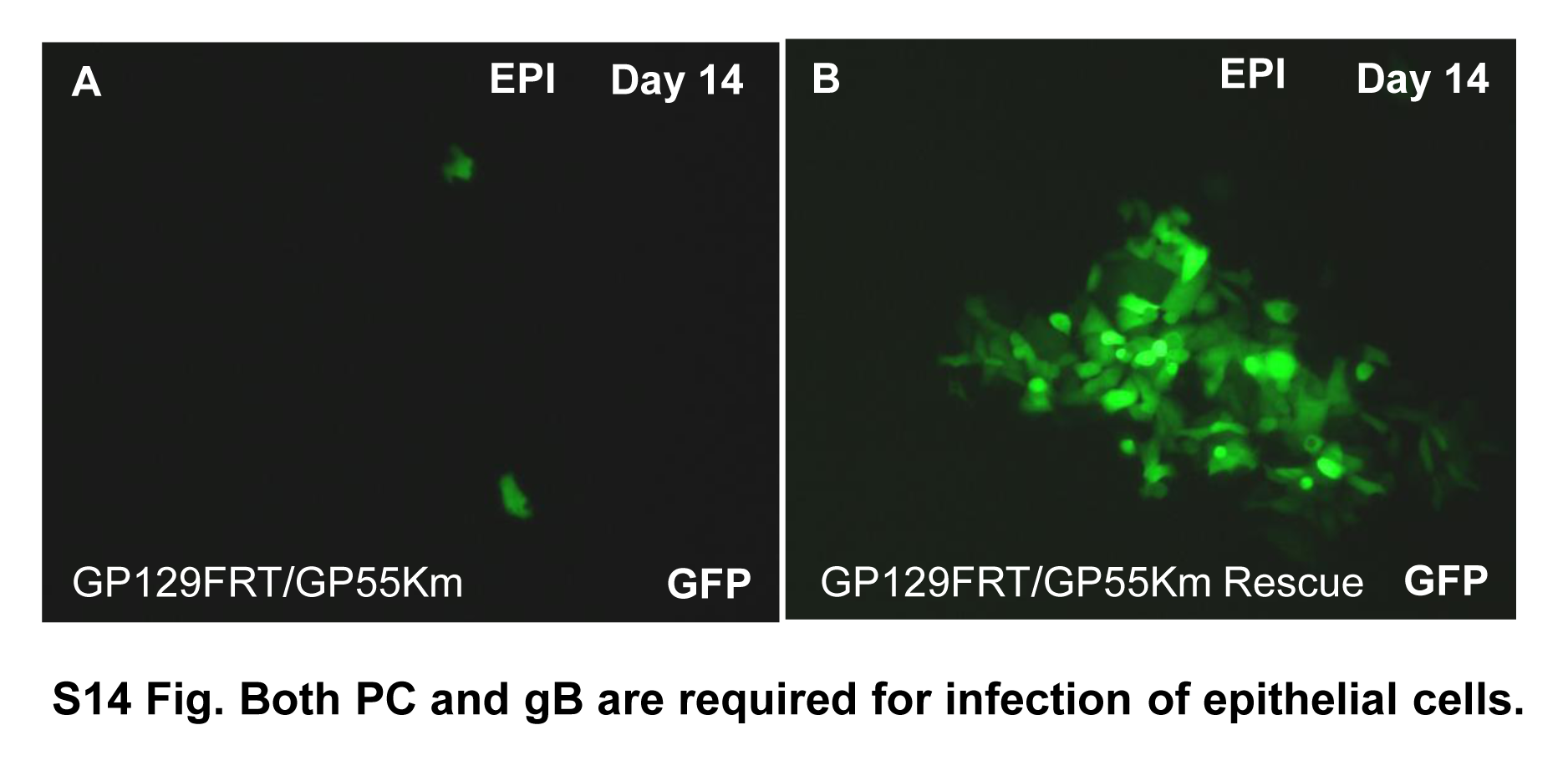

Supplement: S14 Fig — PC+/gO+/ gB negative GPCMV BAC mutant (GP129FRT/GP55Km) transfected onto epithelial cells failed to produce infectious virus (A). Single transfected cells identified by GFP reporter gene expression; gB rescue (GP129FRT/GP55Km rescue). Mutant gB BAC (GP129FRT/GP55km) was co-transfected onto GPL cells with a GP55 rescue fragment to restore epithelial tropism. Virus spread detected by GFP reporter gene expression at day 12 post transfection restored (B). (TIF) [file ppat.1005755.s016.tif]

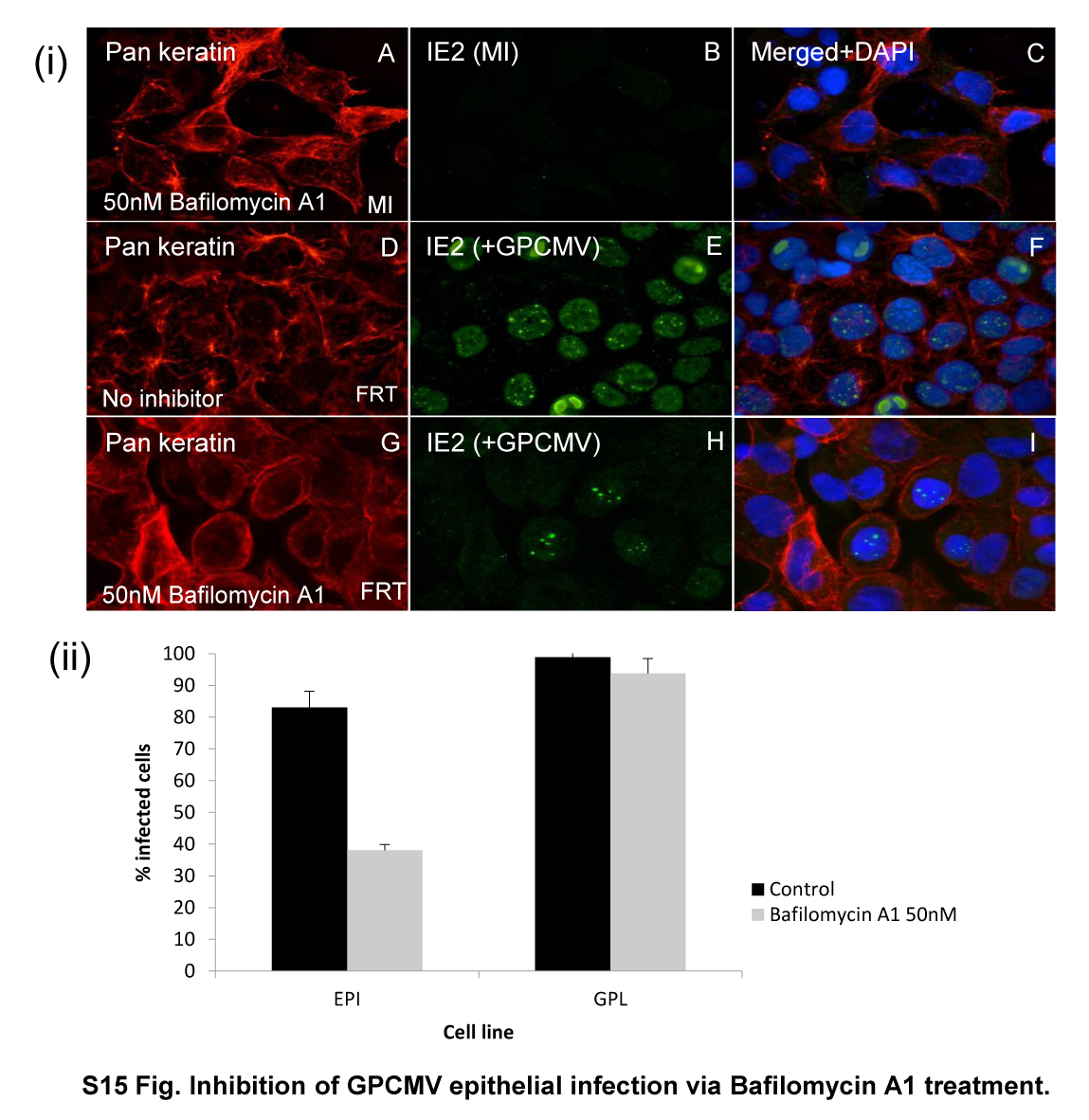

Supplement: S15 Fig — EPI or GPL cells were untreated or pretreated with 50nM bafilomycin A1 for 1 hr prior to GPCMVGP129FRT virus infection (MOI = 1pfu/cell). (i) Virus infection of EPI cells in presence or absence of bafilomycin. Images of random cell fields immunostained for cytokeratin and GPCMV IE2 protein. Images of cells: mock infected EPI cells treated with 50nM bafilomycin A1 (images A-C); untreated EPI cells infected with FRT virus (images D-F); pretreated with 50nM bafilomycin A1(images G-I). Cells immunostained as described in materials and methods: anti-pan-keratin antibody with secondary anti-mouse IgG-TritC (A, D, and G) and anti-IE2 antibody with secondary anti-rabbit IgG-FitC (B, E, and H). Cells were counter stained with DAPI (C, F and I). Images were taken at 40X magnification using spinning disc confocal microscope (Olympus). (ii) Percentage of GPCMV infected cells (GPL or EPI) either bafilomycin treated or untreated. Virus detected by IE2 antigen expression via immunofluorescence assay using anti-IE2 antibody as described in materials and methods. Thirty random fields for each 3 independent experiments per treatment were counted. Statistical analysis was performed with student t-test. (TIF) [file ppat.1005755.s017.tif]
